# PhylDiag: identifying complex synteny blocks that include tandem duplications using phylogenetic gene trees

**DOI:** 10.1186/1471-2105-15-268

**Published:** 2014-08-08

**Authors:** Joseph MEX Lucas, Matthieu Muffato, Hugues Roest Crollius

**Affiliations:** Ecole Normale Supérieure, Institut de Biologie de l’ENS, IBENS, 46 rue d’Ulm, 75005 Paris, France; CNRS, UMR 8197, 75005 Paris, France; Inserm, U1024, 75005 Paris, France; Wellcome Trust Genome Campus, The EMBL-European Bioinformatics Institute, Düsternbrooker Weg 20, CB10 1SD Hinxton, Cambridge UK

**Keywords:** Comparative genomics, Synteny, Synteny block, Segmental homologies, Homology, Gene order, Rearrangement, Ancestral genome, Gene tree

## Abstract

**Background:**

Extant genomes share regions where genes have the same order and orientation, which are thought to arise from the conservation of an ancestral order of genes during evolution. Such regions of so-called conserved synteny, or synteny blocks, must be precisely identified and quantified, as a prerequisite to better understand the evolutionary history of genomes.

**Results:**

Here we describe PhylDiag, a software that identifies statistically significant synteny blocks in pairwise comparisons of eukaryote genomes. Compared to previous methods, PhylDiag uses gene trees to define gene homologies, thus allowing gene deletions to be considered as events that may break the synteny. PhylDiag also accounts for gene orientations, blocks of tandem duplicates and lineage specific de novo gene births. Starting from two genomes and the corresponding gene trees, PhylDiag returns synteny blocks with gaps less than or equal to the maximum gap parameter *gap*_*max*_. This parameter is theoretically estimated, and together with a utility to graphically display results, contributes to making PhylDiag a user friendly method. In addition, putative synteny blocks are subject to a statistical validation to verify that they are unlikely to be due to a random combination of genes.

**Conclusions:**

We benchmark several known metrics to measure 2D-distances in a matrix of homologies and we compare PhylDiag to i-ADHoRe 3.0 on real and simulated data. We show that PhylDiag correctly identifies small synteny blocks even with insertions, deletions, incorrect annotations or micro-inversions. Finally, PhylDiag allowed us to identify the most relevant distance metric for 2D-distance calculation between homologies.

**Electronic supplementary material:**

The online version of this article (doi:10.1186/1471-2105-15-268) contains supplementary material, which is available to authorized users.

## Background

Changes in the order of genes in a genome are caused by two categories of mutational events: genic events, which include de novo gene births, deletions, duplications, and genomic rearrangements, which include chromosome fusions and fissions, segmental translocations or segmental inversions. Synteny blocks are composed of those genes that retain an ancestral organisation despite these events, and one way to understand how genic events and genomic rearrangements affect genome evolution is to identify such synteny blocks. The extremities of synteny blocks also define the positions of breakpoints where rearrangements took place. Precisely defining synteny blocks thus allows, in turn, an accurate definition of breakpoints [1], which has important implications from ancestral genome reconstruction [2] to the understanding of genome mutational processes in healthy and disease states [3]. In addition, it has been shown in eukaryotes that some synteny blocks may be under negative selection due to long-range functional constraints between genes and regulatory elements [4, 5].

Several methods have been developed to identify synteny blocks from extant chromosomes comparisons. In the field of bacterial genome evolution, algorithms tend to focus on the notion of “gene team” [6], which denotes a set of genes that stay in the vicinity of each other with no constraint on gene order. Such methods include TEAM [7], HomologyTeams [8], CCCPart [9], CloseUp [10] and MCMuSeC [11].

However, because gene order conservation in eukaryotes is stronger [12] compared to bacteria, algorithms that infer synteny blocks in eukaryotes tend to account for this extra constraint. GRIMM-synteny [13], i-ADHoRe 3.0 (often just called ADHoRe later) [14–17], DiagHunter [18], LineUp [19], FISH [20], DAGchainer [21], SyMAP [22], ColinearScan [23], Cinteny [24], OrthoCluster [25], Syntenator [26] and Cyntenator [27], MCScan [28] and MCScanX [29], Enredo [30], and DRIMM-Synteny [31] are the main algorithms developed to infer synteny blocks in eukaryotes. Many were applied to model species such as *Arabidopsis thaliana* and rice, among plants, and mammals such as human, mouse, dog and rat, among metazoans. These algorithms can be broadly classified according to their heuristic and features.

Four distinct heuristics are used to infer synteny blocks. The first builds two-dimensional matrices filled with homologies [13, 17, 18, 20, 22, 24]. The algorithms analyse the matrices with procedures that resemble those developed in the field of image analysis.

A second heuristic uses optimisation techniques and dynamic programming [19, 21, 28]. Many of the methods that fall in this category are greedy, although with the benefit of often providing more flexibility. Indeed, the choice of the cost parameters in the objective function, allows the user to accurately account for different synteny block characteristics. A third heuristic is based on a modification of the Smith-Waterman [32] approach [23, 26] while the last type of heuristic relies on graph editing [30, 31].

Some algorithms compare genomes by performing pairwise comparisons of genomes whereas others perform multi-genomes comparisons. Combining pairwise comparisons does not capture the additional significance of genes that are conserved in more than two regions, resulting in under-estimation of cluster significance [33]. Multi-genomes comparisons are especially relevant for highly diverged synteny blocks and Whole Genome Duplication (WGD) analysis. However, multi-genomes comparisons usually require genomes to be reduced to a set of markers shared between all genomes, thus limiting the resolution of the analysis.

The transcriptional orientations of genes on the chromosome are used by some algorithms and provide informations about micro-rearrangements and may contribute to making the correct choice when there are several possibilities to extend a synteny block. In addition, accounting for gene orientations increases the statistical relevance of small synteny blocks, see [Additional file 1: Section 11].

Gene duplications increase the complexity of identifying synteny blocks. Duplications can be dispersed, or in tandem when the two copies are adjacent. Tandem duplications create blocks of tandem duplicates that disrupt local gene adjacencies without strictly breaking the synteny. In order to overcome blocks of tandem duplicates, algorithms may propose to collapse tandem duplicates into one occurrence by remapping their coordinates [17, 20] or by performing ad hoc editions of the graph of adjacencies [30, 31]. WGDs complicate matters further when new genes copies have been randomly inactivated throughout the genome. Yet some algorithms identify highly diverged synteny blocks or double conserved syntenies caused by WGDs [17, 19].

Once an algorithm has returned putative synteny blocks, a statistical validation can assess their relevance given the input data. A putative synteny block is more likely to be found by chance in a comparison involving a large number of homologies than when few homologies are available. A putative synteny block is also less likely to have occurred by chance if it is composed of a large number of ordered adjacent homologies than if it is composed of a few unordered homologies separated by gaps. Statistical validation may involve either a p-value, an e-value or a score. The analytical calculation is not a simple task [8, 33–36] and there is no standard p-value yet established in the field. Simulations are often used to bypass this difficulty, although they are usually time consuming and not very realistic.

To infer a synteny block, each algorithm uses parameters such as the maximum gap *gap*_*max*_ to define the maximum allowed distance between two genes in a synteny block. The *gap*_*max*_ parameter value can be optimised through a theoretical exploration, saving the need to test numerous different values before finding the optimal value [23].

Another important variable is the metric used to allow gaps between genes within a synteny block. Some algorithms use the Diagonal Pseudo Distance [17, 18] whereas others use the Manhattan Distance [13, 20, 22, 24].

Finally, a useful feature is to represent synteny blocks graphically, such as diagonals in a matrix [18], circular views [29] or alignments [14, 29].

Here, we are interested in reconstructing synteny blocks to capture the signals of ancestral gene order and gene orientations in eukaryotic genomes. To this end, we developed PhylDiag, a user-friendly method to identify synteny blocks between two genomes using reconstructed phylogenetic gene trees. The full evolutionary history of each ancestral gene is taken into account in the form of those phylogenetic gene trees, which include in particular gene losses, duplications, 1:1 but also 1:many and many:many homology relationships. All PhylDiag parameters can either be set automatically or be specified by the user. A p-value calculation provides a statistical basis to select significant blocks and a utility provides graphical representations of identified synteny blocks. Users may also chose among several metrics to allow gene gaps within a synteny block. PhylDiag accounts for tandem duplications and gene orientations, and is thus able to accurately identify small synteny blocks. Among algorithms that already account for gene order and gene orientations, only i-ADHoRe 3.0, FISH and Enredo also handle tandem duplications, although they do not use gene trees reconstructions. Here we compare PhylDiag to i-ADHoRe 3.0 [14] (version i-ADHoRe 3.0.2a) using both real data and simulations.

By introducing the concepts of “tandem blocks” and “homology packs”, PhylDiag overcomes the disruption of gene adjacencies caused by blocks of tandem duplicates. As in other existing methods, PhylDiag allows gaps between genes within synteny blocks up to a customizable maximum gap parameter, and thus bypasses small genic indels (insertions and deletions) and annotation errors. In this study, we also benchmark different metrics used to allow these gaps within a synteny block on simulated data, and show that the choice of the metric has a direct impact on performances.

## Methods

After providing basic definitions, we describe the PhylDiag algorithm, which consists of four main parts. First, PhylDiag filters extant genomes. Second, PhylDiag rewrites the genomes from lists of genes to lists of tandem blocks. Third, PhylDiag extracts synteny blocks as diagonals with no gaps by considering the order and orientations of tandem blocks on the chromosomes and then merge these diagonals as long as merges do not generate gaps longer than *gap*_*max*_. Finally, PhylDiag computes a p-value to remove diagonals that are likely to be produced by chance rather than being a signature of an ancestral gene order. Before performing these tasks PhylDiag also calculates a recommended value for the maximum gap *gap*_*max*_ to free the user from testing multiple values before finding the appropriate one.

### Basic notations and definitions

#### Genomic conventions

*S* is a species. Given two species *S*_*a*_ and *S*_*b*_, LCA(*S*_*a*_,*S*_*b*_) is the Last Common Ancestor of *S*_*a*_ and *S*_*b*_. A species *S*_*a*_ has a genome *G*_*a*_ composed of chromosomes.  is a chromosome of *G*_*a*_ with *N*_*a*_ oriented genes g_*a*,*k*_. The chromosome is chosen to be ordered from g_*a*,1_ to g_*a*,*N*_ and not the reverse, thus defining a reference orientation. The orientation of a gene is determined by the orientation of transcription into RNA, and the orientation of g_*a*,*k*_, denoted *o*(g_*a*,*k*_), is equal to +1 if transcription is performed in the same direction as  otherwise *o*(g_*a*,*k*_)= −1. A sub-list of *c*_*a*_ is often denoted *c*_*a*_[*i*_*s*_→ *i*_*e*_] where *i*_*s*_ (respectively *i*_*e*_) is the index of the starting (respectively ending) gene in the sub-list.

#### Synteny block, intuitive definition

Intuitively (a formal definition is given in ‘Synteny block, formal definition’) we define a Synteny Block (sb, plural sbs) between two species *S*_*a*_ and *S*_*b*_ as a set of neighbouring genes with gene content, gene order and gene orientations conserved during the evolution from LCA(*S*_*a*_,*S*_*b*_) to *S*_*a*_ and *S*_*b*_. Two genes are neighbours if they are separated by less than a user-defined parameter *gap*_*max*_. During evolution we consider that a set of neighbouring genes remains a synteny block until:

− a chromosomal rearrangement creates a breakpoint within the sb and changes the order or the orientations of genes
− the gap between any two neighbouring genes, caused by gene insertions and/or gene deletions, exceeds *gap*_*max*_ genes (see the formal definition of *gap*_*max*_ in ‘Synteny block, formal definition’ for the choice of the type of gene insertions or gene deletions that may break the synteny)

An ancestral sequence of genes remains a sb even if tandem duplications occur within the synteny block.

#### Gene family and homology

The evolution of a gene can be represented by a rooted binary tree called a gene tree. The root of a gene tree is the first ancestral gene, the nodes correspond to events of speciations or duplications that occurred during the evolutionary history of the descending genes, and the leaves of the gene tree correspond to extant genes originating from the first gene.

Two genes are homologs if they are in the same gene tree. Two genes are orthologs if they are in the same gene tree and if their last common event is a speciation. Two genes are paralogs if they are in the same gene tree and if their last common event is a duplication. The homology relationship between two genes g_*a*_ and g_*b*_ is denoted . A homology relation defines classes of homologs, called families. An issue in comparative genomics is to define gene families and gene trees. Sequence comparison algorithms provide measures (such as BLASTP [37] scores) that make it possible to quantify the similarity between two sequences which may, in turn, be used to cluster genes that show high similarity, thus defining gene families. Gene families can then be organised in phylogenetic gene trees using a vast choice of tree reconstruction methods. Here, we use gene trees from Ensembl [38], built using the TreeBest pipeline [39]. Since in this study we are interested in finding synteny blocks conserved from LCA(*S*_*a*_,*S*_*b*_) to *S*_*a*_ and *S*_*b*_, we pruned all gene trees to define a gene family as a set of genes that come from a unique gene of LCA(*S*_*a*_,*S*_*b*_). Families are defined with these genes, so that two genes are in the same family if and only if they come from the same ancestral gene of LCA(*S*_*a*_,*S*_*b*_). We note that, depending on the purpose of the analysis, PhylDiag offers the possibilty to prune gene trees at an ancestor that precedes LCA(*S*_*a*_,*S*_*b*_), so that more paralogy relationships are included in the gene family, see [Additional file 1: Section 1].

**Figure 1:**
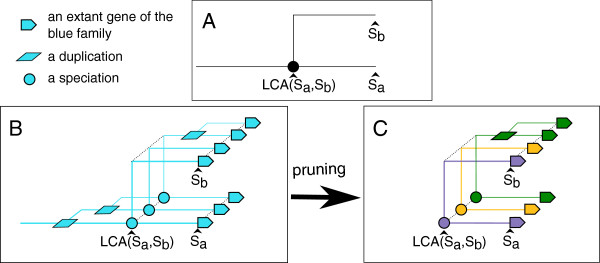
**Definition of gene families based on gene tree pruning.** Figure **A** represents a species tree with two extant species *S*_*a*_ and *S*_*b*_, and their last common ancestor LCA(*S*_*a*_,*S*_*b*_). Figure **B** represents a gene tree within the species tree. This gene tree is represented in simple 3D schema for better visualisation. In a gene tree, squares represent duplication events and circles represent speciation events. Figure **C** shows how the original gene tree of figure B is pruned in order to define families that correspond to a unique gene of LCA(*S*_*a*_,*S*_*b*_).

Considering the species tree of Figure 1A and the original gene tree of Figure 1B, the Figure 1C describes how we pruned the original gene trees to define our families. Ultimately, the roots of the gene trees correspond to a unique gene of LCA(*S*_*a*_,*S*_*b*_).

### Step 1: Filter extant genomes

When comparing two species *S*_*a*_ and *S*_*b*_, the first step of PhylDiag is to propose a filtering of extant genomes. There are two filters:

− *InBothSpecies* removes genes that have no homolog in the other genome. This only retains genes that previous algorithms call “anchor genes” and it is the classical way of filtering extant genomes. This filter is well suited for finding functional clusters of genes.
− *InCommonAncestor* removes genes that arose de novo specifically after LCA(*S*_*a*_,*S*_*b*_). The removed genes are those that have no ancestral gene in LCA(*S*_*a*_,*S*_*b*_) and they are called “lineage specific genes”. The selective removal is possible using the pre-computed phylogenetic gene trees. This step is equivalent to retaining “anchor genes”, but here, using gene trees, the procedure also keeps genes that have lost their ortholog in the other species because of a deletion since LCA(*S*_*a*_,*S*_*b*_). This filtering is well suited for reconstructing ancestral gene orders.

Both filtering get rid of the noise introduced by lineage specific genes. PhylDiag using the *InBothSpecies* filter does not consider ancestral gene deletions as events that break the synteny whereas PhylDiag using the *InCommonAncestor* filter does consider ancestral gene deletions as events that break the synteny.

It may also be advantageous in some specific cases of functional studies of synteny blocks to avoid filtering extant genomes thus considering de novo births of lineage specific genes as events that break the synteny.

Depending on the desired purpose, PhylDiag offers the possibility to easily choose between no filtering at all, the *InBothSpecies* filter or the *InCommonAncestor* filter. Since in this study we are interested in reconstructing the ancestral gene order, the *InCommonAncestor* filter is applied and extant genomes should now be considered to only be composed of genes that have an ancestral gene in LCA(*S*_*a*_,*S*_*b*_).

### Step 2: Build the matrix of homology packs

Extracting sbs conserved in *G*_*a*_ and *G*_*b*_ corresponds to extracting sbs for each comparison of chromosomes *c*_*a*_ of *G*_*a*_ and *c*_*b*_ of *G*_*b*_. Indeed, genes in two different chromosomes, if they were in synteny before, have been separated by a chromosomal rearrangement and the synteny is broken anyway. Thus it is justified to limit the search to pairs of chromosomes rather than pairs of genomes.

#### Tandem blocks, an abstraction of genes

In a chromosome, under a parsimonious reasoning, homologous and adjacent genes are tandem duplicates. Here, we refer to such blocks as “tandem block”. Formally, a tandem block (tb, plural tbs) of a chromosome *c* is an uninterrupted sub-list of *c* that contains paralogous genes. For instance, if the 3 paralogous genes *g*_4_,*g*_5_ and *g*_6_ are in an uninterrupted row in *c*, the corresponding tb is the sub-sequence *c*[ 4→6]=[ g_4_,g_5_,g_6_]. The size of a tb is equal to the number of tandem gene copies that it contains, for instance the last tb has a size 3. A gene which has no tandem duplicate is in a tb of size 1. By convention tbs are always maximum, i.e. a given tb cannot be contained within another tb. Like genes, tbs also have an orientation. However, in a tb, tandem duplicates may or may not all have the same orientation. When they all share the same orientation, the tb itself is oriented with the same orientation as the orientation of the genes thus, either *o*(tb)=+1 or *o*(tb)=−1. When tandem duplicates have different orientations, the orientation of the tb is considered to be *unknown*, and .

It is possible to rewrite chromosomes as a unique ordered list of oriented tbs. For instance  can be rewritten  where *n*_*a*_ is the number of tbs in *c*_*a*_. *n*_*a*_≤*N*_*a*_ and *n*_*a*_=*N*_*a*_ if and only if there is no tandem duplicate in *c*_*a*_.

A tandem block tb_*a*_ of *S*_*a*_ is said to be in a homology relation with a tandem block tb_*b*_ of *S*_*b*_ if the genes of the two tbs are in the same family. We will also say that in this case tb_*a*_ and tb_*b*_ are homologs or even that tb_*a*_ and tb_*b*_ are homologous tandem blocks. Using the same notation as for genes,  means that tb_*a*_ and tb_*b*_ are homologs. If tb_*a*_ and tb_*b*_ are homologs, they share a Last Common Ancestral gene in LCA(*S*_*a*_,*S*_*b*_) and we note LCAg(tb_*a*_,tb_*b*_) the Last Common Ancestral gene of tb_*a*_ and tb_*b*_. LCAg(tb_*a*_,tb_*b*_) is defined as soon as it is observed that . Of note, two homologous tandem blocks tb_*a*_ and tb_*b*_ are not necessarily of the same size if deletions or tandem duplications took place specifically in the branches of *S*_*a*_ or *S*_*b*_ after LCA(*S*_*a*_,*S*_*b*_).

#### Matrix of homologies

The classic Matrix of Homologies  of two chromosomes  and  is defined such that:


Where *g*_*a*_• *g*_*b*_ is the “sign” of the homology of *g*_*a*_ and *g*_*b*_

A MH can be represented as an array of values equal to +1,−1 or 0. Non-0 values correspond to homologies.

#### Homology packs, an abstraction of homologies

A Homology Pack (hp, plural hps) is the set of homology relationships between the tandem duplicates of two homologous tandem blocks tb_*a*_ (in *c*_*a*_) and tb_*b*_ (in *c*_*b*_). A hp is always maximum, i.e. a hp cannot be contained within another hp. Graphically, a hp appears as a rectangle of non-0 values in a MH. Each hp has a last common ancestral gene in LCA(*S*_*a*_,*S*_*b*_) denoted LCAg(hp) and equal to LCAg(tb_*a*_,tb_*b*_). Tandem duplications generate vertical, horizontal, or rectangular hps in a MH, making it difficult to identify sbs as diagonals. However, the rewriting of a chromosome in a way that collapses these hps to unique values in the MH, as described above, greatly simplifies this problem. Indeed, once *c*_*a*_ and *c*_*b*_ are rewritten as ordered lists of tbs, it becomes possible to define a matrix whose non-0 values correspond to hps of the two chromosomes *c*_*a*_ and *c*_*b*_.

#### Matrix of homology packs

Given that *c*_*a*_ is rewritten in  and *c*_*b*_ is rewritten in , we introduce the Matrix of Homology Packs  of the two chromosomes  and  defined such that:


Whith tb_*a*_∙tb_*b*_ the “sign” of the hp of tb_*a*_ and tb_*b*_

In other words, the matrix construction is the same as for the MH of *c*_*a*_ and *c*_*b*_, with tbs instead of genes and hps instead of gene homologies. The only difference is that while genes always have a known orientation, tbs can have *unknown* orientations that generate hps with signs equal to . Similarly, the MHP can be represented as an array of values equal to  or 0. Non-0 values correspond to hps. The X-axis corresponds to *c*_*a*_ ordered from tb_*a*,1_ to  and the Y-axis corresponds to *c*_*b*_ ordered from tb_*b*,1_ to . With this convention MHP[0,0] corresponds to the bottom-left corner, MHP[*n*_*a*_,0] corresponds to the bottom-right corner, MHP[0,*n*_*b*_] corresponds to the top-left corner and MHP[*n*_*a*_,*n*_*b*_] corresponds to the top right corner of the array.

[Additional file 1: Section 2] gives a graphical representation of the transition between the MH and the MHP via rewriting chromosomes with tbs.

#### Distances and gaps

The “gap between two tbs” on the same chromosome is the number of tbs between them.

The “distance between two tbs” is equal to the gap between these two tbs *plus one*. Thus two adjacent tbs are at a distance one from each other.

As in definition 2.1 in [35], a set of tbs forms a “chain” with gaps ≤ *gap*_*max*_ if all consecutive tbs are separated by gaps ≤ *gap*_*max*_ tbs.

**Figure 2:**
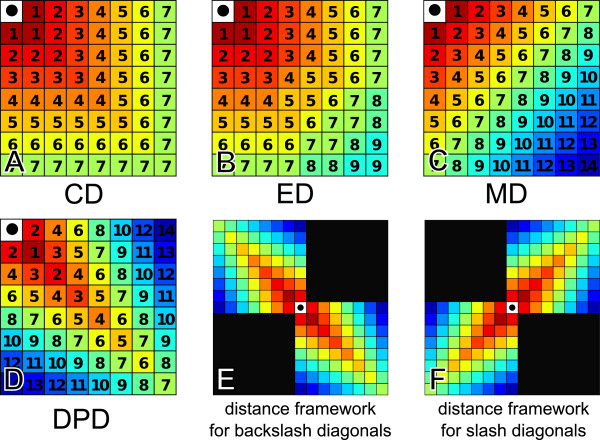
**Distance metrics and frameworks used for the distance calculation.** Figures **A**, **B**, **C** and **D** represent the metrics available in PhylDiag. Distance values are calculated starting from the black dot. The warmer the colour, the closer the point from the black dot. Considering that the user chose the DPD, when backslash diagonals are merged, the backslash framework of figure **E** is used for the distance calculation. For a slash diagonal merge, the framework of figure **F** is used. Frameworks would have been built in the same fashion if another metric had been chosen.

Now, given a MHP, we define the “distance between two hps” as the 2D-distance between hps coordinates which depends on a distance metric. Several distance metrics can be used in PhylDiag: the Euclidean Distance (ED), the Chebyshev Distance (CD), the Manhattan Distance (MD), or the Diagonal Pseudo Distance (DPD) (Figure 2). Equations for each distance metric can be found in [Additional file 1: Section 3]. The CD yields the maximum of the distances on *c*_*a*_ and *c*_*b*_, the ED yields the classical geometric distance, the DPD yields smaller distances between hps sitting close to the diagonal axis and therefore tends to provide a higher distance as the distance from the diagonal axis increases. In contrast, the MD tends to yield smaller distances between hps sitting close to the vertical and horizontal axis.

We define the “gap between two hps” as the distance between these hps *minus one*, thus a gap between two hps depends on the distance metric used. A gap of 0 between two hps means that there is no gap and this corresponds to a distance equal to 1. Given a maximum gap *gap*_*max*_, a set of hps forms a “cluster” if no gap between them is longer than *gap*_*max*_.

### Step 3: Extract putative synteny blocks as consistent diagonals

In the following section, we define the notion of consistent diagonals in a MHP and we formally define synteny blocks. Then, we explain how synteny blocks generate consistent diagonals in MHPs, and we describe how PhylDiag extracts consistent diagonals. Because some consistent diagonals may be due to chance, we next describe how they are validated as synteny blocks after succeeding a statistical test.

#### Diagonals

In a MHP, a list of *m* hps [MHP[*x*_*k*_,*y*_*k*_]]_*k*∈[0,*m*−1]_ forms a:

− “slash” diagonal if .
− “backslash” diagonal if .

In both cases, *x*_*k*_ (respectively *y*_*k*_) is the index of the homologous tb on *c*_*a*_ (respectively *c*_*b*_) corresponding to the *k*^*th*^ hp. In a MHP, a “slash” diagonal is thus a list of non-0 cells that goes up according to a direction from bottom-left to top-right and a “backslash” diagonal is a list of non-0 cells that goes down according to a direction from top-left to bottom-right. A “diagonal” is either a slash diagonal or a backslash diagonal. A diagonal with gaps ≤*gap*_*max*_ is a diagonal where all consecutive hps are separated by gaps ≤*gap*_*max*_.

We define a “strict” diagonal as a diagonal that has no gap between its hps. Thus, *m* hps form a strict slash diagonal if the list of *m* hps can be written [ MHP[ *s*_*a*_+*k*,*s*_*b*_+*k*]]_*k*∈[0,*m*−1]_. Similarly, *m* hps form a strict backslash diagonal if the list of *m* hps can be written [ MHP[ *s*_*a*_+*k*,*s*_*b*_−*k*]]_*k*∈[0,*m*−1]_. In both cases, (*s*_*a*_,*s*_*b*_) is the position of the first hp of the diagonal.

We also define a “consistent” diagonal as a diagonal composed of hps with signs consistent with hps order:

− either a slash diagonal only composed of hps with signs equal to either +1 or
− or a backslash diagonal only composed of hps with signs equal to either −1 or

In addition, we consider that the distance between two diagonals corresponds to the distance between their closest extremities.

#### Synteny block, formal definition

We formally define a “synteny block” of *m* tbs with gaps ≤ *gap*_*max*_ of a comparison of two genomes *G*_*a*_ and *G*_*b*_, as a chain of *m* tbs with gaps ≤ *gap*_*max*_ that, during the evolution from LCA(*S*_*a*_,*S*_*b*_) to *S*_*a*_ and *S*_*b*_, remains a chain of *m* tbs with gaps ≤ *gap*_*max*_. Within a synteny block, tbs order is conserved and tbs orientations either remain conserved or change from a known to an unknown orientation. Synteny blocks are chosen maximal, i.e. not included in another synteny block.

In addition we define a “strict synteny block” as a synteny block with no gaps between tbs (*gap*_*max*_=0). In [Additional file 1: Section 4], we show that a strict synteny block generates a strict and consistent diagonal in a MHP. Following the reasoning in [Additional file 1: Section 4], using the CD distance metric, it is also possible to show that a synteny block with gaps ≤ *gap*_*max*_ generates a consistent diagonal with gaps ≤ *gap*_*max*_. However, using the ED, MD or the DPD distance metrics, a synteny block with gaps ≤ *gap*_*max*_ may generate a consistent diagonal with gaps >*gap*_*max*_, although every consistent diagonal with gaps ≤ *gap*_*max*_ always represents a putative synteny block with gaps ≤ *gap*_*max*_. It should be noted that, given the CD distance metric and a *gap*_*max*_, our definition of a diagonal is similar to the definition 4.1 of a “max-gap cluster” in [35] with constraints on gene order and gene orientations.

#### Extract strict consistent diagonals

Algorithm 1 describes how PhylDiag finds strict and consistent diagonals of hps in the MHP. First, chromosomes are rewritten with tbs and the MHP is built. Then the MHP is scanned from left to right and from bottom to top. Algorithm *findDiagType* in [Additional file 1: Section 5], sets the diagonal type at the beginning of a strict and consistent diagonal extraction using the sign of the first hp if the sign is known or using the position of the second hp if there is a second hp.

Strict and consistent diagonals are recorded as chains of ordered and oriented (whenever it is possible) ancestral genes. By convention the orientation of an ancestral gene LCAg(tb_*a*_,tb_*b*_) is chosen equal to the orientation of tb_*a*_. However, if the orientation of tb_*a*_ is *unknown*, the orientation of LCAg(tb_*a*_,tb_*b*_) may still be inferred using the diagonal type of the current diagonal and a known orientation of tb_*b*_, see Equation 1.
1

##### Algorithm 1 1 ***extractSbs*(*c***_***a***_**,*c***_***b***_**)**



#### Merge strict consistent diagonals

Once strict diagonals have been returned, it is advantageous to merge diagonals which have the same diagonal type, as long as their extremities are in close proximity. Depending on the allowed gap size *gap*_*max*_, a limited number of errors of annotation and indels are thus allowed, and longer sbs are found that still reflect an ancestral arrangement of genes. It should be noted that this step possibly introduces micro-inversions within gaps of a diagonal, which will however always remain shorter than *gap*_*max*_ tbs. As we will see, the choice of the distance metric used to merge diagonals is crucial to limit or allow such micro-inversions, see [Additional file 1: Section 14].

The merging process is simple: diagonals are merged iteratively, starting by those separated by the shortest gap to those separated by the longest gap, as long as the gap remains below *gap*_*max*_. For a given diagonal extremity, more than one other extremity may be situated at exactly the same distance. In this case, PhyDiag chooses to fuse the diagonals that maximise the number of hps in the diagonal that results from the fusion.

As described in the introduction, the DPD is used in ADHoRe and DiagHunter whereas the MD is used in GRIMM-Synteny, FISH, Cinteny and SyMAP. Although the CD and the ED have never been used to our knowledge in the context of synteny block inference we still included them in the benchmark presented in the ‘Results’ section.

**Figure 3:**
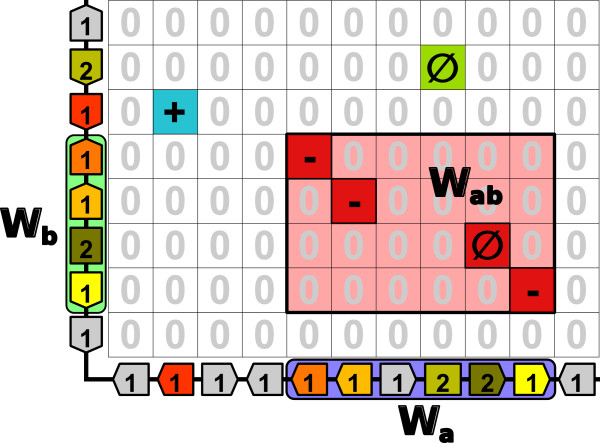
**Example of a merge between two diagonals.** Two chromosomes, *c*_*a*_ of *n*_*a*_= 11 tbs and *c*_*b*_ of *n*_*b*_= 8 tbs, are compared. The number in each tb is its size, arrows indicate tbs orientations and if a tb is represented as a rectangle it means that it has an *unknown* orientation. The MHP contains *n*_*ab*_= 6 hps. During step 2, PhylDiag finds two strict backslash diagonals and 2 single hps. Each strict diagonal contains 2 hps. If *g* *a* *p*_*max*_= 4, during the diagonal merging process, diagonals separated by a distance of 2 to *g* *a* *p*_*max*_+1=5 are merged. Consider that the user chooses the DPD metric, when reaching a distance of 5, two possible fusions are theoretically possible, one between the two extremities of the strict backslash diagonals and another fusion between the single hp (coordinates [ 2,6]) and the extremity (coordinates [5,5]) of the leftmost strict diagonal. However since the sign of the single hp is not consistent with the diagonal type of the left-most diagonal, the second fusion is not performed. Around the resulting consistent diagonal (in red) three windows are drawn: *W*_*a*_ (purple) on *c*_*a*_ contains 6 tbs and *W*_*b*_ (green) on *c*_*b*_ contains 4 tbs, and at last the window *W*_*ab*_ (pink) contains 6 × 4 cells and 4 hps. These windows are useful in section ‘Step 4: Statistical validation of consistent diagonals as synteny blocks’ for the p-value calculation.

Figure 3 shows an example of a merge between two strict backslash diagonals spaced by a distance 5 if the user chose the DPD, 4 if the user chose the MD and 3 if the user chose the CD or the ED.

Given a maximum gap *gap*_*max*_, users should be aware that, with reference to the formal definition of sbs given in section ‘Synteny block, formal definition’, choosing another distance metric than the CD may return non-maximum sbs in the MHP. Another reason that may lead to non-maximum sbs may come from the fusion of diagonals. As mentioned before, if during the fusion process more than one diagonal extremity is available to extend the current diagonal, PhylDiag choses the extremity of the longest diagonal. However, it may be that fusing with a shorter one ultimately would lead to a longer diagonal once the iterative fusion process is complete.

In Algorithm 1, the merging process is encapsulated in the function *mergeDiags* that takes a list of strict and consistent diagonals and returns a list of consistent diagonals with gaps ≤ *gap*_*max*_.

### Step 4: Statistical validation of consistent diagonals as synteny blocks

We compare two chromosomes, *c*_*a*_ and *c*_*b*_. *c*_*a*_ has a length of *n*_*a*_ tbs, *c*_*b*_ has a length of *n*_*b*_ tbs and the comparison involves *n*_*ab*_ hps. During the comparison, PhylDiag returns many consistent diagonals that correspond to putative synteny blocks, each characterized by its number of hps, its window *W*_*ab*_ and the maximum gap *g* between its tbs (note that *g* ≠ *gap*_*max*_). Figure 3 shows an example of a consistent diagonal of 4 hps contained in the window *W*_*ab*_ with a maximum gap *g*=2 tbs reached on *c*_*a*_. The window *W*_*ab*_ has a size 6 × 4. The chromosomal windows *W*_*a*_ and *W*_*b*_ are the projections of *W*_*ab*_ on each chromosome. *W*_*a*_ has a length of *l*_*a*_= 6 tbs and *W*_*b*_ has a length of *l*_*b*_= 4 tbs. As in previous works [33–35], here distances and gaps between hps are calculated with the Chebyshev Distance metric which allows the most relaxed and method-independent sb definition.

A given consistent diagonal is a statistically significant signature of a sb if it cannot be obtained from a random distribution of tbs (null-hypothesis) up to a fixed probability threshold *α*. This is equivalent to selecting consistent diagonals that are unlikely to be the result of chance, which we wish to quantify here by a probability, a p-value.

We calculate the p-value of each consistent diagonal in five steps. Considering a consistent diagonal of *m* hps contained in a window *W*_*ab*_ of size *l*_*a*_× *l*_*b*_ with a maximum gap between hps equal to *g*, the probability that such a consistent diagonal (or an even more improbable consistent diagonal with gaps ≤ *g*) arises by chance is denoted *pVal*(*m*,*g*,*l*_*a*_,*l*_*b*_,*n*_*ab*_,*n*_*a*_,*n*_*b*_). To compute this value we first compute *p*_*d*_(*k*,*l*_*a*_,*l*_*b*_,*n*_*ab*_,*n*_*a*_,*n*_*b*_) the probability of obtaining exactly *k* hps in the window *W*_*ab*_, knowing the MHP density in terms of non-0 values. We next compute *p*_*g*,2D_(*k*,*g*,*l*_*a*_,*l*_*b*_), the probability that *k* hps in *W*_*ab*_ are spaced with gaps ≤ *g*, knowing that there is at least *k* hps in *W*_*ab*_. We also calculate *p*_*o*,*o*_(*k*) the probability that *k* hps have consistent order and signs. By summing and multiplying these probabilities in an appropriate manner we calculate *p*_*w*_(*m*,*g*,*l*_*a*_,*l*_*b*_,*n*_*ab*_,*n*_*a*_,*n*_*b*_), the probability corresponding to a window sampling search. Finally, we use the former probability to compute the p-value *pVal*(*m*,*g*,*l*_*a*_,*l*_*b*_,*n*_*ab*_,*n*_*a*_,*n*_*b*_) corresponding to a whole genome comparison. The formulas of the two first probabilities are based on [33, 35] respectively and the passage from *p*_*w*_ to the *pVal* is based on [34]. Here we combine these probabilities and add a last probability, *p*_*o*,*o*_(*k*), to account for tbs order and orientations.

#### Probability accounting for the density

Using the reasoning of [33], in a MHP of size *n*_*a*_× *n*_*b*_ without dispersed paralogy (see Discussion), involving *n*_*ab*_ hps, the probability of obtaining exactly *k* hps in a window *W*_*ab*_ of size *l*_*a*_× *l*_*b*_ is:
2

The subscript *d* stands for *density* because this probability takes into account the density of the MHP. The demonstration of this formula is in [Additional file 1: Section 6].

#### Probability accounting for the maximum gap between hps

Using the reasoning of [35], the probability that *k* marked tbs (in any order) form a chain with gaps ≤ *ganywhere* within a window composed of *l* tbs is:
3

Where *w*_*kg*_= *k*+(*k*−1)*g* is the maximum length of a chain containing *k* tbs with a maximum gap *g*, and
4

the number of ways of arranging *k* tbs so that they form a chain with gaps shorter or equal to *g* anywhere within a window of *l* tbs even if *w*_*kg*_>*l*+1, to address edge effects.

Thus, knowing that *W*_*ab*_ contains at least *k* hps, the probability that *W*_*ab*_ contains *k* marked hps spaced with gaps ≤ *g* is:
5

#### Probability accounting for hps order and signs

Then, if *k* hps are close enough, the probability that they form a consistent slash diagonal with gaps ≤ *g* is:
6

Where  and *P*(*sign* = *s*) is the probability that one hp sign equals *s*, this probability calculation is explained in [Additional file 1: Section 7].  is the probability that *k* homologous tbs of chromosome *c*_*b*_ have the *same* order as the corresponding *k* homologous tbs of chromosome *c*_*a*_ and  is the probability that the *k* signs of the hps are consistent with a slash diagonal. *p*_*backslash*_(*k*) is defined similarly.

Thus, if *k* hps are close enough, the probability that they form a consistent diagonal with gaps ≤ *g* is:
7

The subscript *o*,*o* stands for consistent tbs *Order* and tbs *Orientations*. The demonstration of the *p*_*o*,*o*_ formula can be found in [Additional file 1: Section 8].

#### Probability for a window sampling scenario

Now, in a MHP of size *n*_*a*_× *n*_*b*_ without dispersed paralogy (see Discussion), involving *n*_*ab*_ hps, the probability that in a window *W*_*ab*_ of size *l*_*a*_× *l*_*b*_ there is *at least* one consistent diagonal containing *at leastm* hps spaced with gaps ≤ *g* is:
8

The subscript *w* stands for *Window* because this probability corresponds to a window sampling [34] scenario. Only varying parameters are shown in the right-hand side of the equation in the preceding formula. This formula is explained in [Additional file 1: Section 9].

#### Probability for a whole chromosome comparison

Finally, since PhylDiag performs a whole chromosome comparison, it is not possible to use the probability of a window sampling method that would underestimate the probability to find a consistent diagonal by a factor of *O*(*n*_*a*_*n*_*b*_). Thus, relying on the reasoning of section 4.2 of [34] we adjust the former probability to compute the probability corresponding to a whole chromosome comparison.

In a MHP of size *n*_*a*_× *n*_*b*_ containing *n*_*ab*_ hps without dispersed paralogy (see Discussion), the probability of finding *at least* one window *W*_*ab*_ of size *l*_*a*_× *l*_*b*_ containing *at least* a consistent diagonal of *at leastm* hps spaced by gaps ≤ *g* can be approximated by:
9

where  is the number of windows of width *l*_*a*_ and height *l*_*b*_ in the MHP such that no window overlap with any other window. The underlying assumption of this formula is justified in [Additional file 1: Section 10] and examples of calculation are performed in [Additional file 1: Section 11].

In Algorithm 1, the statistical validation is encapsulated in the function *statisticalValidation* that takes a list of consistent diagonals as input and returns statistically validated sbs.

### Estimation of a recommended maximum gap parameter

All algorithms designed to identify synteny blocks use a maximum gap parameter (*gap*_*max*_) to allow gaps in sbs. However, the user may find it difficult to estimate the optimal value for this parameter. In order to avoid guessing or multiple trials before finding the optimal *gap*_*max*_ value, PhylDiag uses the dependency between the probability of finding a consistent diagonal of *m* hps spaced by gaps ≤ *gap*_*max*_ and the *gap*_*max*_ value. The complete reasoning used to calculate the recommended maximum gap parameter can be found in [Additional file 1: Section 12].

### Viewer

**Figure 4:**
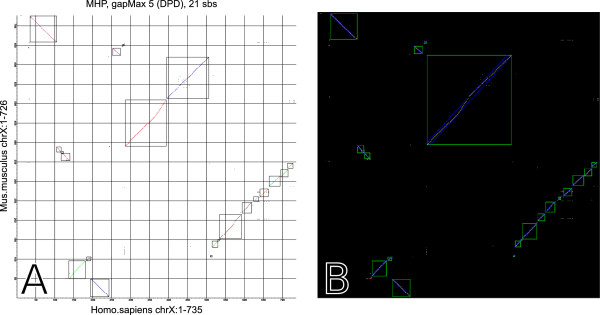
**Representations of a comparison between the human and the mouse X chromosomes, produced from the same input data by PhylDiag (A) and i-ADHoRe 3.0.2a (B).** The maximum gap parameter *g* *a* *p*_*max*_ is equal to 5 and the merging process used the DPD metric in both cases. In figure A each axis displays explicitly the paths to the files containing the relevant genome data, the name of the chromosome and the chromosomal window range. As in ADHoRe sbs identified by PhylDiag are surrounded by a rectangle and each sb is drawn in a specific colour. In figure B blue dots represent a confidence interval around each sb drawn in yellow. The long synteny block in the middle of the ADHoRe MHP is in two parts in the PhylDiag MHP because the two extremities are spaced by a distance higher than 5 using the DPD metric. By default ADHoRe has a special feature using extremities of diagonals during its merging process, however this feature cannot be deactivated which may lead to undesired merges.

PhylDiag includes a utility to visualise the MHP of a pairwise comparison of chromosomes with colours and surrounding black rectangles for sbs recognition. This viewer writes a vectorial image allowing an infinite zoom on details with no pixelisation. Figure 4A shows an example of the viewer during the comparison of the human X chromosome with the mouse X chromosome. If more information about a region of the MHP is required, a zoom can be performed by specifying the desired chromosomal regions. If these are small enough, more information is shown, such as hps signs, oriented tbs on each axis, the size of each tb and colours for homology recognition. Grey tbs represent tbs that do not have hps in the MHP, but they have hps elsewhere in the pairwise comparisons of genomes, in another pairwise comparison of chromosomes. Figure 3 was produced with the viewer and shows such informations. The user may also visualise the MH, for example to study the genic composition of a tb.

### Implementation

The complete algorithm has been implemented in Python. Pairwise comparisons of chromosomes are performed in parallel since they are independent. In Algorithm 1, the MHP matrix is stored considering that it is a sparse matrix to reduce memory usage and the merging process is optimised. Combinations in probability formulas are computed using Pascal’s rule and dynamic programming. On a single 3,0 GHz processor with 32 Gb RAM, loading the data in memory requires 3 seconds, and the running time for the pairwise analysis of the Human and Mouse genomes requires less than 3 seconds. Without any optimisation of the memory allocations the peak of RAM consumption is 221 Mb, thus a standard personal computer can run PhylDiag.

## Results

To evaluate the performances of PhylDiag, we performed a comparative analysis with i-ADHoRe 3.0 [14], a state-of-the-art algorithm used in many recent studies. To make comparisons possible however, we used a version of the program provided by the authors. Indeed, i-ADHoRe 3.0 first rewrites genomes in tbs like PhylDiag, but allows a user-defined “tandem_gap” between genes in a tb. In version 3.0, the minimal tandem_gap is 2, and it is not possible to set the tandem_gap to 0, as in PhylDiag. In the version provided (i-ADHoRe 3.0.2a) this option is enabled.

When ADHoRe compares two chromosomes, it first generates “baseclusters” which correspond to PhylDiag’s sbs. ADHoRe uses the DPD metric to build baseclusters containing gaps ≤“gap_size” in the matrix of homologies. ADHoRe also uses the “prob_cutoff” parameter for the statistical filtering and a last parameter is the “q_value”, a real value between 0 and 1, indicating the minimum *r*^2^ (a measure for the linearity of baseclusters in the matrix of homologies) that a cluster should display.

### Comparison with i-ADHoRe 3.0.2a on real data

In a first comparison, we provided the same input based on real genomic data to PhylDiag and ADHoRe. We used the human genome (*G*_*h*_) and the mouse genome (*G*_*m*_) of Ensembl v72. As explained in section ‘Gene family and homology’, families correspond to genes that are descended from a unique gene of LCA(*S*_*h*_,*S*_*m*_)= Euarchontoglire.

**Figure 5:**
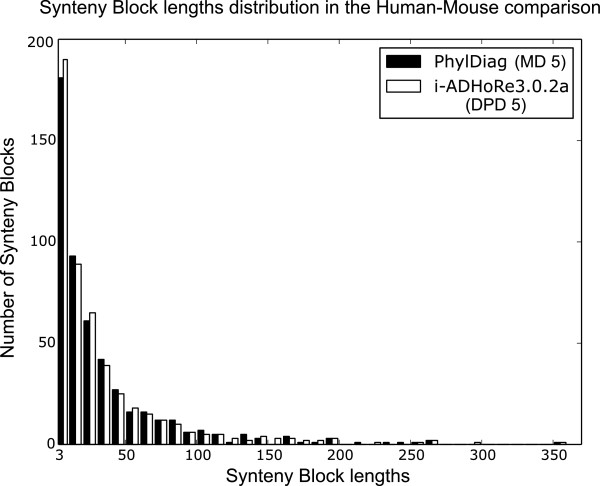
**Synteny block lengths distribution as computed by PhylDiag (black) and ADHoRe (white).** Each bin has a width of 10 units of length (hps), apart from the first bin that contains sb lengths from 3 to 10 hps. ADHoRe uses the DPD distance metric whereas PhylDiag uses the MD distance metric.

PhylDiag computes a recommended *gap*_*max*_ of 5 tbs for the human-mouse comparison. We therefore set a *gap*_*max*_ parameter of 5 and we chose a probability threshold *α* = 1 × 10^−3^ for PhylDiag. i-ADHoRe 3.0.2a was set with tandem_gap=0, gap_size=5, prob_cutoff=1×10^−3^ and q_value=0.9 (the default value). Figure 5 compares the distributions of synteny block lengths of ADHoRe and PhylDiag using the MD distance metric. The two distributions are not different from each other (Mann-Whitney U test: pval = 0.9791), and show that neither methods suffer from strong biases in over or under detection of synteny blocks in a given size range. Of note, PhylDiag returned 17 significant sbs of 2 hps out of 175 consistent diagonals of 2 hps. These are not shown in Figure 5 because ADHoRe does not report sbs of size 2. PhylDiag statistically validated all consistent diagonals containing more than 2 hps as significant synteny blocks.

### Comparison with i-ADHoRe 3.0.2a on simulated data

Our simulator first designs an ancestral genome *G*_*anc*_ with a user defined number of genes and chromosomes. The lengths of chromosomes in *G*_*anc*_ are expressed in number of genes, and are determined randomly. Simulated evolution gives rise to the two extant genomes *G*_*a*_ and *G*_*b*_ of two extant species. The simulator performs genic events, which include de novo gene births, deletions, duplications (tandem and dispersed), and genomic rearrangements, which include chromosome fusions and fissions, segmental translocations or segmental inversions. The evolutionary scenario is calibrated so as to fit the known evolution of the human and the mouse genome from the Euarchontoglire genome using phylogenetic gene tree reconstructions from Ensembl Compara version 72. See [Additional file 1: Section 13] for a more detailed description of the Simulator.

We performed 100 simulations of the evolution of the human and the mouse genome, and analysed them with PhylDiag and ADHoRe to identify sbs. The PhylDiag merging process was performed with the 4 different distance metrics (ED,CD,DPD and MD). For ADHoRe the DPD is the only distance metric available. As in the comparison with real data, since the simulation is calibrated to fit real evolutionnary rates, the recommended *gap*_*max*_ found by PhylDiag is still 5. Results of PhylDiag with a *gap*_*max*_=5 and ADHoRe with *gap*_*size*= 5 are shown in Table 1.

**Table 1:** **Results of synteny block identification with PhylDiag and i-ADHoRe3.0.2a, both using a** ***gap***_***max***_ ***= 5***

Algorithm	PhylDiag (without sbs of 2 hps)				ADHoRe
distance	ED	CD	DPD	MD	DPD
coverage	98.71%	98.74%	97.02%	98.55%	96.55%
N50	44.69	46.62	32.66	37.33	31.71
**Analysis without gene orientations**					
sensitivity	94.99%	95.06%	92.26%	94.32%	91.68%
specificity	99.92%	99.85%	99.90%	99.98%	99.83%
**Analysis with gene orientations**					
sensitivity	94.20%	94.26%	91.56%	93.54%	88.56%
specificity	99.08%	99.01%	99.13%	99.15%	96.43%

Since ADHoRe only returns sbs containing at least 3 hps, we only consider PhylDiag’s sbs containing at least 3 hps.

*Coverage* is the fraction of the number of gene families (each family corresponds to a single ancestral gene of Euarchontoglire) contained in sbs over the total number of ancestral genes conserved in both the simulated human genome and the simulated mouse genome. *N50* is the length of the sb such that all sbs of greater lengths represent 50*%* of the ancestral genes contained in sbs. *Sensitivity* is the fraction of the number of correctly inferred ancestral adjacencies over the total number of ancestral genes conserved in both the simulated human genome and the simulated mouse genome. *Specificity* is the fraction of the number of correctly inferred ancestral adjacencies over the total number of inferred ancestral adjacencies, false inferences included.

Specificity and sensitivity are calculated twice: first by ignoring gene orientations (an inferred adjacency between two genes is considered correct if both genes are adjacent in *G*_*anc*_ even if their relative orientation is different compared to the ancestral relative orientation), and second by taking gene orientations into account (to be correct an inferred adjacency must contain genes with a relative orientation that is the same as in the *G*_*anc*_).

Results show that PhylDiag with the DPD, and ADHoRe obtain similar results when we do not consider gene orientations during the analysis. Interestingly, simply using the ED, the CD or the MD metrics allows PhylDiag to achieve better sensitivity and specificity than ADHoRe (Mann-Whitney U test on sensitivity % and specificity % using the MD in PhylDiag and DPD in ADHoRe over 100 simulations: pval ≤ 2.2e-16 and pval ≤ 2.2e-16 respectively). In addition, as soon as gene orientations are considered in the analysis, PhylDiag improves substantially, in part because of Equation 1.

## Discussion

We have compared PhylDiag to i-ADHoRe 3.0, a state-of-the-art algorithm including advanced features which are not present in PhylDiag, including the possibility to identify sbs in the “twilight zone”, i.e. sbs highly diverged or separated by a WGD, where many gene deletions may have occurred. ADHoRe uses “profiles” across more than two genomes to identify poorly conserved sbs, for example due to long divergence times. These features were not exploited here because unlike ADHoRe, PhylDiag only performs pairwise comparisons of genomes since our primary interest is to identify sbs in closely related species.

We explored different distance metrics to measure distances in matrices of homology, and found that the DPD used in ADHoRe, which favours fusions of diagonals along ±45° axes in the MHP (Figures 2D, 2E and 2F), is not optimal. This has been discussed previously [23] and the simulations clearly show that exploring first laterally (i.e. vertically and horizontally), as with the MD (Figure 2C), improves results. Merging diagonals with the DPD distance metric allows more small inversions within sbs gaps while considering that genic/segmental indels and incorrect annotations break the synteny more easily than with the MD. Conversely, merging diagonals with the MD metric gives priority to lateral directions and this allows more small genic/segmental indels and annotation errors within sbs gaps and considers that inversions break the synteny more easily than with the DPD, see [Additional file 1: Section 14]. Interestingly the unusual ED or CD distance metrics also show improved results over the DPD (Table 1). It should be noted that a given distance may cover a different number of cells in the MHP depending on the metric chosen. For instance 9 cells are covered within a distance value of 3 with the MD whereas 7 cells are covered within the same distance value of 3 with the DPD (Figures 2C and 2D). Although this bias may play a role in the results, on chromosomes, gaps between tbs involved in pairs of chains corresponding to sbs are always smaller or equal to *gap*_*max*_ independently of the metric chosen. Thus comparing metrics is fair. Finally, contrary to ADHoRe, PhylDiag can return sbs containing 2 hps if their p-value is under the p-value threshold of the user.

PhylDiag includes a new statistical validation to estimate the probability that a putative sb may be due to chance. Unlike other tests, it accounts for gene orientations, thus providing increased sensitivity. It also accounts for tandem duplications but ignores the possibility that duplicate gene copies may be dispersed. Neglecting dispersed duplicates underestimates the p-values of sbs and the significance of sbs are thus overestimated. However models considering gene families exist [8, 23] and in a future version it might be advantageous to implement the p-value proposed in [36], even if the calculation is based on an unrealistic assumption that all gene families are of fixed size. Nevertheless the error in the p-value calculation in PhylDiag is likely to be small for closely related species. For instance the analysis of phylogenetic trees described here shows that only 2.4*%* of tbs are dispersed duplicates in the human genome (3.2*%* in mouse) using our family definition (section ‘Gene family and homology’).

The p-value used by PhylDiag is relative to a comparison of two chromosomes, and therefore assumes that random consistent diagonals might arise based on the number of tbs and hps relevant to the two chromosomes only. In contrast, a global (i.e. genome wide) threshold *α* is chosen to distinguish significant sbs from non-significant sbs. This inconsistency represents an area of further development, in order to better account for heterogeneous densities of hps depending on which chromosomes are being compared.

## Conclusion

PhylDiag is designed around a heuristic-independent formal definition of synteny blocks. Its implementation and benchmarking using real and simulated data allowed us to rank 2D-distance metrics in terms of sensitivity and specificity, and to evaluate its performance in comparison with ADHoRe. Results show that the DPD distance metric yields the poorest performances when identifying synteny blocks, both with ADHoRe and PhylDiag. In contrast, PhylDiag highlights the interesting sensitivity-specificity trade-off achieved by the MD distance metric, closely followed by the CD and the ED distance metrics. Compared to ADHoRe and other algorithms that infer synteny blocks, the definition of gene families in PhylDiag is based on gene trees. Most notably, this feature offers the opportunity to precisely group extant genes into families that descend from a unique gene in the last common ancestor of the two species being compared. Furthermore, a meticulous attention to tandem duplicates and gene orientations allow PhylDiag to reach a high resolution in the analysis of rearrangements, down to single gene inversion. Finally, the statistical validation of putative synteny blocks filters out putative false positives due to randomly convergent gene order. PhylDiag is a software for synteny block inference that benefits from extensive parameters, including *gap*_*max*_, distance metric, p-value threshold, filtering of extant genomes and ancestor for the gene family definition. Their values can be set by PhylDiag (default values are based on previous benchmarks or set automatically based on the data) or set by the user. These features, together with post-processing graphical analysing tools and printed statistics (number of tandem duplicates in extant genomes, number of dispersed duplicates, number of homologies involved in the pairwise comparison) contribute to making PhylDiag a user-friendly method to find synteny blocks.

## Availability and requirements

**Project name**: PhylDiag **Project home page**: https://github.com/DyogenIBENS/PhylDiag**Operating system(s)**: Platform independent **Programming language**: Python **Other requirements**: Python 2.7 or higher **License**: GNU GPL v3 or later, and the CeCiLL v2 license in France **Any restrictions to use by non-academics**: No

## Electronic supplementary material

Additional file 1: **Supplementary Data.** (PDF 793 KB)
