# Supplementary material for: PhylDiag: identifying complex synteny blocks that include tandem duplications using phylogenetic gene trees
Source: BMC Bioinformatics. 2014 Aug 8;15(1):268. doi: 10.1186/1471-2105-15-268 (PMC4155083; doi:10.1186/1471-2105-15-268)
Supplement: Supplementary file 1 — Additional file 1: Supplementary Data. (PDF 793 KB) [file 12859_2014_6554_MOESM1_ESM.pdf]

Supplementary Data

PhylDiag : identifying complex synteny blocks that include tandem duplications using phylogenetic gene trees

Joseph MEX Lucas, Matthieu Muffato and Hugues Roest Crollius

1 Different ways of defining gene families

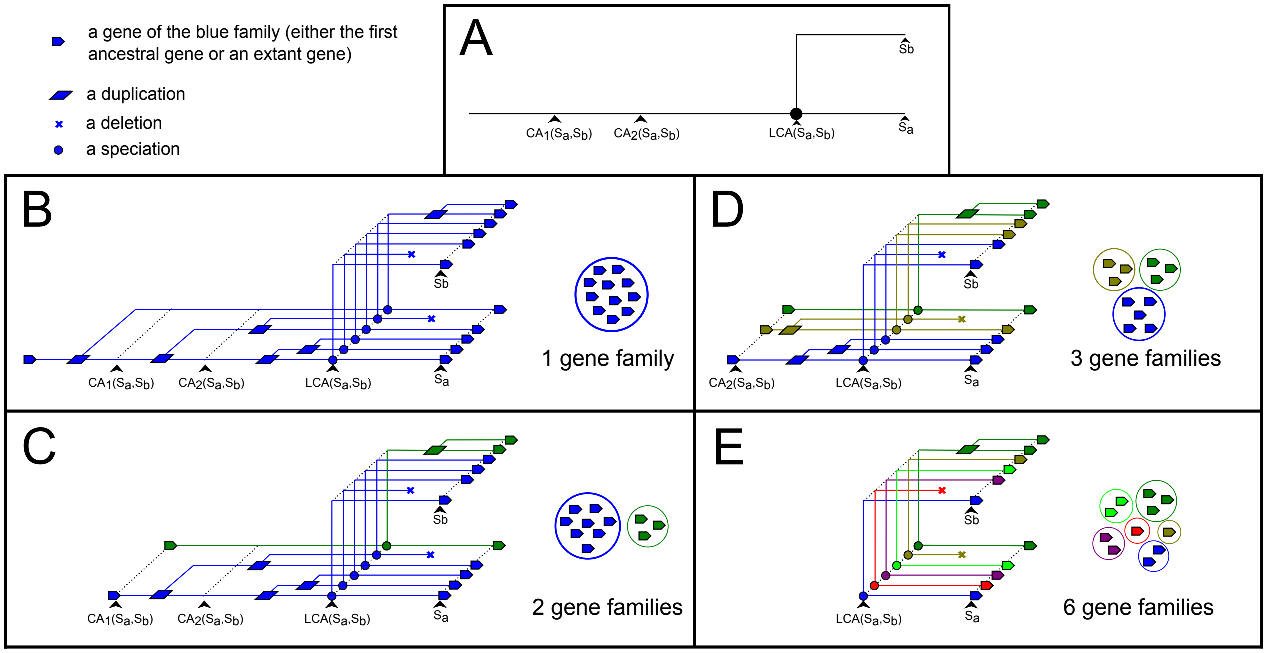

Figure S1: **Different ways of defining gene families.** Figure A represents a species tree with two extant species S_a and S_b. $\mathsf{LCA}(S_a, S_b)$ is the last common ancestor of species S_a and S_b, $\mathsf{CA}_1(S_a, S_b)$ is a common ancestor of S_a and S_b, and $\mathsf{CA}_2(S_a, S_b)$ is another common ancestor of S_a and S_b that lived more recently than $\mathsf{CA}_1(S_a, S_b)$. Figure B represents a gene tree within the species tree. This gene tree is represented in simple 3D schema for better visualisation. In a gene tree, squares represent duplication events, circles represent speciation events and crosses represent deletion events. Figure C shows how the original gene tree of figure B is pruned in order to define families that correspond to a unique gene of $\mathsf{CA}_1(S_a, S_b)$. Figure D shows how the original gene tree of figure B is pruned in order to define families that correspond to a unique gene of $\mathsf{CA}_2(S_a, S_b)$. Finally Figure E shows how the original gene tree of figure B is pruned in order to define families that correspond to a unique gene of $\mathsf{LCA}(S_a, S_b)$. Figures show that the more recent is the ancestor used for the pruning the more families.

2 From the MH to the MHP by rewriting chromosomes with tbs

Figure S2a is an example of a matrix of homologies. Along the X-axis and the Y-axis, arrows represent oriented genes. This MH corresponds to a chromosome c_a of 8 genes on the X-axis and a chromosome c_b

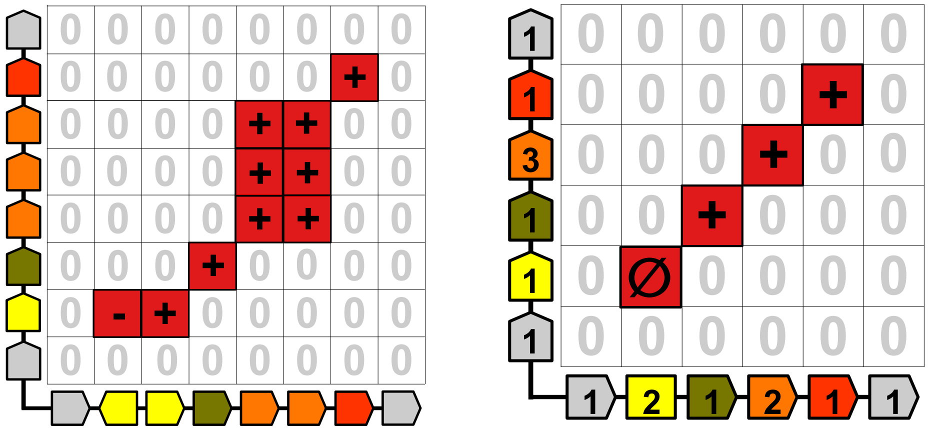

(a) Matrix of Homologies (MH) (b) Matrix of Homology Packs (MHP)

Figure S2: **A matrix of homologies and the corresponding matrix of homology packs**

of 8 genes on the Y-axis. Genes with the same colour are homologs except for grey genes which have no homologies in the current MH but which have homologies in other pairwise comparisons of chromosomes. Filled cells represent homologies. Homologies of the same colour belong to the same sb. The symbol in a homology represents $\mathsf{g}_a \bullet \mathsf{g}_b$, the sign of the homology, where g_a and g_b are the two homologous genes. For convenience, a sign $+1$ is denoted $+$ and a sign -1 is denoted $-$.

c_a can be written $[\mathsf{g}_{a,k}]_{k\in[1,8]}$, where $\mathsf{g}_{a,1}$ is the leftmost gene of c_a and $\mathsf{g}_{a,8}$ is the rightmost gene of c_a. Adjacent homologous genes are considered as tandem duplicates thus the second and third genes of c_a, $\mathsf{g}_{a,2}$ and $\mathsf{g}_{a,3}$, are tandem duplicates. $\mathsf{g}_{a,2}$ has an orientation equal to -1 whereas $\mathsf{g}_{a,3}$ has an orientation equal to $+1$. c_a can be rewritten with 6 tbs, $c_a = [\mathsf{tb}_{a,k}]_{k\in[1,6]}$, where $\mathsf{tb}_{a,1}$ is the leftmost tb and $\mathsf{tb}_{a,6}$ is the rightmost tb. Starting from the left, $\mathsf{tb}_{a,2}$, the second tb of c_a, has an *unknown* orientation, all the other tbs have an orientation equal to $+1$.

In the MH, rectangles of non-0 values represent hps. In this example there are 4 hps. The first hp has a size 2×1, the second hp has a size 1×1, the third hp has a size 2×3 and the last hp has a size 1×1. $\mathsf{tb}_{a,4} = c_a[5 \to 6]$ is in a homology relation with $\mathsf{tb}_{b,4} = c_b[4 \to 6]$. Thus the corresponding hp is the submatrix $\mathsf{MH}[5 \to 6, 4 \to 6]$. This hp is said to have a size 2×3, with 2 the size of $\mathsf{tb}_{a,4}$ and 3 the size of $\mathsf{tb}_{b,4}$.

Figure S2b is the corresponding MHP of the MH of figure S2a after rewriting the chromosomes with tbs. Along the X-axis and the Y-axis, arrows represent oriented tbs. The rectangle on the X-Axis represents a tb with an *unknown* orientation. The values in the arrows are the sizes of each tbs. The symbol in a hp represents $\mathsf{tb}_a \bullet \mathsf{tb}_b$, the sign of the hp, where tb_a and tb_b are the two homologous tbs. For convenience, a sign $+1$ is denoted $+$ and a sign -1 is denoted $-$ whereas an *unknown* sign is denoted $\varnothing$. The bottom-most and left-most hp corresponds to a tb of size 2 (on the X-axis) with an *unknown* orientation in a homology relation with a tb of size 1 with an orientation equal to $+1$ (on the Y-axis). Thus the sign of this hp is *unknown*, i.e. equal to $\varnothing$. Going top and right, the third hp corresponds to a tb of size 2 (on the X-axis) in a homology relation with a tb of size 3 (on the Y-axis). Both tbs have an orientation equal to $+1$. Thus the sign of the corresponding hp is $+1$. All hps have a sign equal to $+1$ except for the first hp which has a sign equal to $\varnothing$.

3 Distance metric formulas

If (x_0, y_0) and (x_1, y_1) are the coordinates of two positions in the MHP, depending on the metric used, the distances between these two positions are given by the formulas:

$$
\begin{aligned}
d_{\mathsf{CD}}((x_0,y_0),(x_1,y_1)) &= max(|x_1 - x_0|, |y_1 - y_0|) \\
d_{\mathsf{ED}}((x_0,y_0),(x_1,y_1)) &= \left\lceil \sqrt{(x_1 - x_0)^2 + (y_1 - y_0)^2} \right\rceil \\
d_{\mathsf{MD}}((x_0,y_0),(x_1,y_1)) &= |x_1 - x_0| + |y_1 - y_0| \\
d_{\mathsf{DPD}}((x_0,y_0),(x_1,y_1)) &= 2max(|x_1 - x_0|, |y_1 - y_0|) - min(|x_1 - x_0|, |y_1 - y_0|)
\end{aligned}
$$

Where $[x]$ is the nearest integer of x. CD stands for Chebyshev Distance metric, ED for Euclidean Distance metric, MD stands for Manhattan Distance metric and DPD stands for Diagonal Pseudo Distance metric. It is easy to construct figure S3 with these formulas.

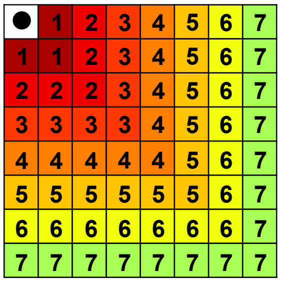

(a) Chebyshev Distance (CD)

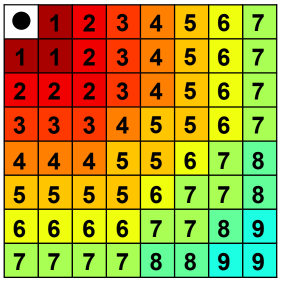

(b) Euclidean Distance (ED)

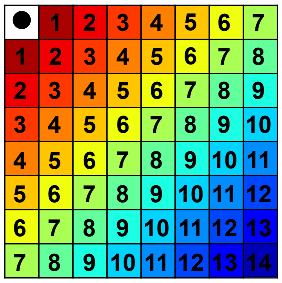

(c) Manhattan Distance (MD)

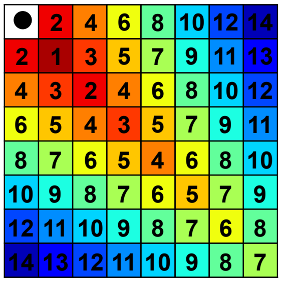

(d) Diagonal Pseudo Distance (DPD)

Figure S3: **Distance metrics available in PhylDiag.** Distance values are computed from the black dot. The warmer the colour, the closer the point from the black dot.

4 Strict consistent diagonals are putative strict synteny blocks

We will demonstrate that extracting putative strict sbs (containing no gaps between tbs) of c_a and c_b is equivalent to extracting strict and consistent diagonals of hps in the related MHP. Let $[\mathsf{g}_i]_{i\in[s,s+l-1]}$ be an uninterrupted ancestral sequence of l tbs in $\mathsf{LCA}(S_a, S_b)$, each tb is a unique gene. If this sequence of tbs is a strict sb, the ancestral sequence remains an uninterrupted sequence of tbs up to the two compared species, furthermore, within the sb, tbs order is conserved and tbs orientations either remain conserved or change from a known to an unknown orientation. Therefore the sb is present in a chromosome c_a of S_a and is also present in a chromosome c_b of S_b. Without loss of generality, we arbitrarily choose the reference order of the tbs of the sb to be the same as in c_a, so $\mathsf{g}_{s+k}\mathcal{H}\mathsf{tb}_{a,s_a+k}$ $\forall k \in [0, l-1]$. Depending on the choice of the order of the tbs in c_b, the order of the syntenic tbs in c_b may thus be in the same order as the syntenic tbs in c_a, or in the reverse order. Thus two cases must be treated (cf figure S4):

- The order of the syntenic tbs in c_b is conserved in the *same* order as the syntenic tbs in c_a so there is a row $[\mathsf{tb}_{a,i}]_{i\in[s_a,s_a+l-1]}$ in c_a and a row $[\mathsf{tb}_{b,i}]_{i\in[s_b,s_b+l-1]}$ in c_b that verify $\forall k \in [0, l-1]$

$$\mathsf{g}_{s+k} = \mathsf{LCAg}(\mathsf{tb}_{a,s_a+k}, \mathsf{tb}_{b,s_b+k})$$

so

$$\mathsf{tb}_{a,s_a+k}\ \mathcal{H}\ \mathsf{tb}_{b,s_b+k}$$

and

$$\mathsf{MHP}[s_a+k, s_b+k] \neq 0.$$

This corresponds to a strict slash diagonal in the MHP.

Furthermore, when the tbs $[g_i]_{i\in[s,s+l-1]}$ do not evolve from a known to an unknown orientation, the tbs conserve their orientations within the sb, i.e. relatively to the choice of the order of tbs in the

sb, from g_s to g_{s+l-1}. Therefore the orientation of tb_{b,s_b+k} relatively to $\overrightarrow{\mathsf{tb}_{b,s_b}\mathsf{tb}_{b,s_b+l-1}}$ is the same as the orientations of tb_{a,s_a+k} relatively to $\overrightarrow{\mathsf{tb}_{a,s_a}\mathsf{tb}_{a,s_a+l-1}}$. $\overrightarrow{\mathsf{tb}_{a,s_a}\mathsf{tb}_{a,s_a+l-1}}$ is the same orientation as the reference orientation of c_a and $\overrightarrow{\mathsf{tb}_{b,s_b}\mathsf{tb}_{b,s_b+l-1}}$ is the *same* orientation as the reference orientation of c_b, thus, when we consider the orientations of tbs on their respective chromosomes, $\forall k \in [0, l-1]$

$$o(tb_{a,s_a+k}) = o(\mathsf{tb}_{b,s_b+k}) \text{ or } \varnothing$$
$$o(tb_{b,s_b+k}) = o(\mathsf{tb}_{a,s_a+k}) \text{ or } \varnothing,$$

so

$$tb_{a,s_a+k} \bullet \mathsf{tb}_{b,s_b+k} = +1 \text{ or } \varnothing$$

and the diagonal is composed of hps with signs equal to either +1 or ∅,

$$\mathsf{MHP}[s_a + k, s_b + k] = +1 \text{ or } \varnothing.$$

It is thus a strict and consistent slash diagonal.

- The order of the syntenic tbs in c_b is conserved in the *reverse* order compared to the syntenic tbs in c_a so there is a row $[\mathsf{tb}_{a,i}]_{i\in[s_a,s_a+l-1]}$ in c_a and a row $[\mathsf{tb}_{b,i}]_{i\in[s_b-l+1,s_b]}$ in c_b that verify $\forall k \in [0, l-1]$

$$\mathsf{g}_{s+k} = \mathsf{LCAg}(\mathsf{tb}_{a,s_a+k}, \mathsf{tb}_{b,s_b-k})$$

so

$$\mathsf{tb}_{a,s_a+k}\ \mathcal{H}\ \mathsf{tb}_{b,s_b-k}$$

and

$$\mathsf{MHP}[s_a + k, s_b - k] \neq 0.$$

This corresponds to a strict backslash diagonal in the MHP.

Furthermore, when the tbs $[g_i]_{i\in[s,s+l-1]}$ do not evolve from a known to an unknown orientation, the tbs conserve their orientations within the sb, i.e. relatively to the choice of the order of tbs in the sb, from g_s to g_{s+l-1}. Therefore the orientation of tb_{b,s_b+k} relatively to $\overrightarrow{\mathsf{tb}_{b,s_b}\mathsf{tb}_{b,s_b-l+1}}$ is the same as the orientations of tb_{a,s_a+k} relatively to $\overrightarrow{\mathsf{tb}_{a,s_a}\mathsf{tb}_{a,s_a+l-1}}$. $\overrightarrow{\mathsf{tb}_{a,s_a}\mathsf{tb}_{a,s_a+l-1}}$ is the same orientation as the reference orientation of c_a but now $\overrightarrow{\mathsf{tb}_{b,s_b}\mathsf{tb}_{b,s_b-l+1}}$ is the *reverse* orientation compared to the reference orientation of c_b, thus, when we consider the orientations of tbs on their respective chromosomes, $\forall k \in [0, l-1]$

$$o(tb_{a,s_a+k}) = -o(\mathsf{tb}_{b,s_b-k}) \text{ or } \varnothing$$
$$o(tb_{b,s_b-k}) = -o(\mathsf{tb}_{a,s_a+k}) \text{ or } \varnothing,$$

so

$$tb_{a,s_a+k} \bullet \mathsf{tb}_{b,s_b-k} = -1 \text{ or } \varnothing$$

and

$$\mathsf{MHP}[s_a + k, s_b - k] = +1 \text{ or } \varnothing.$$

It is thus a strict and consistent backslash diagonal.

We demonstrated that a strict sb conserved from $\mathsf{LCA}(S_a, S_b)$ to S_a and S_b generates a strict and consistent diagonal in the MHP of a chromosome c_a in G_a and a chromosome c_b in G_b, either a strict and consistent slash diagonal or a strict and consistent backslash diagonal.

It may be, in theory, that a strict and consistent diagonal in a MHP does not correspond to a sb if some tbs, brought in adjacent positions, generate strict and consistent diagonals by chance. However the statistical validation of PhylDiag ensures that when such a case is highly probable the strict and consistent diagonal is not considered as a signature of a strict synteny block. That is why we consider that a strict and consistent diagonal is only a *putative* strict synteny block. A strict and consistent diagonal is considered as a synteny block if it passes the statistical validation.

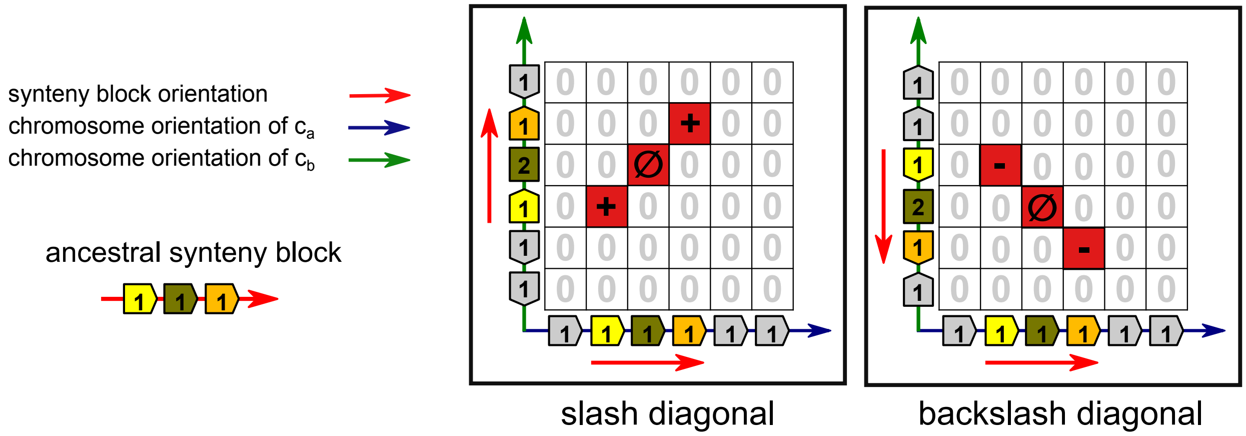

Figure S4: **Provenance of the distinction between slash and backslash diagonals.** In the leftmost MHP, the order of the chromosome c_b defines a reference orientation that is in the *same* orientation as the orientation of the synteny block. With this order of tbs in the chromosome c_b, the synteny block yields a strict and consistent *slash* diagonal, that goes up according to a direction from bottom-left to top-right. In the rightmost MHP, the order of the chromosome c_b defines a reference orientation that is in the *reverse* orientation compared to the orientation of the synteny block. With this order of tbs in the chromosome c_b, the synteny block yields a strict and consistent *backslash* diagonal, that goes down according to a direction from top-left to bottom-right.

5 Algorithm *findDiagType*

findDiagType sets the diagonal type at the beginning of a strict and consistent diagonal extraction using the sign of the first hp if the sign is known or using the position of the second hp if their is a second hp. If two known diagonal types (either slash or backslash) are possible, the slash type is chosen by default. By convention the algorithm gives an orientation *unknown* to a single hp not involved in a strict diagonal.

Algorithm 1 *findDiagType*(MHP, (i, j))

inputs
  MHP: Matrix of Homology Packs
  (i, j): coordinates of the first hp of a diagonal in MHP
if MHP$[i, j] \neq \varnothing$ **then**
  $diagType = \begin{cases} \text{slash}, & \text{if MHP}[i,j] = +1 \\ \text{backslash}, & \text{if MHP}[i,j] = -1 \end{cases}$
else
  if MHP$[i+1, j+1] = +1$ or $\varnothing$ **then**
    //the sign of the next top-right hp is consistent with a slash diagonal
    $diagType \leftarrow$ slash
  else if MHP$[i+1, j-1] = -1$ or $\varnothing$ **then**
    //the sign of next bottom-right hp is consistent with a backslash diagonal
    $diagType \leftarrow$ backslash
  else
    $diagType \leftarrow unknown$
return $diagType$

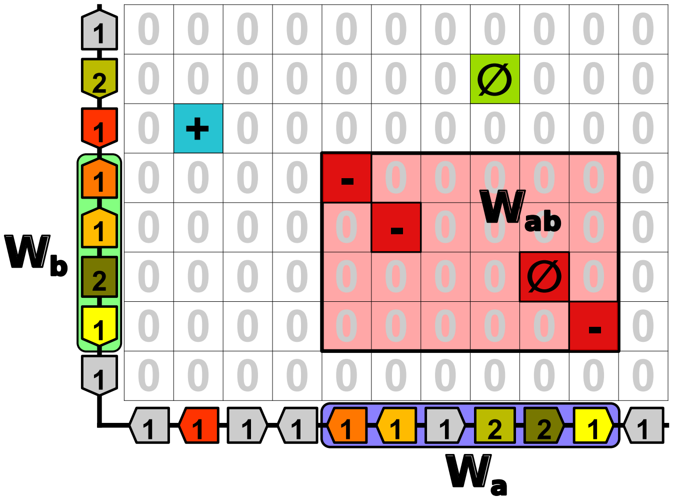

Figure S5: **Characterisation of a consistent diagonal in the MHP.** Two chromosomes, c_a of $n_a = 11$ tbs and c_b of $n_b = 8$ tbs, are compared. The MHP contains $n_{ab} = 6$ hps. During step 2, after the merging process PhylDiag returns a consistent diagonal of $m = 4$ hps contained in the window W_{ab} (pink) with a maximum gap $g = 2$ tbs reached on c_a. The window W_{ab} has a size 6×4. The chromosomal windows W_a (purple) and W_b (green) are the projections of W_{ab} on each chromosome. W_a has a length of $l_a = 6$ tbs and W_b has a length of $l_b = 4$ tbs.

6 Demonstration of the p_d formula

Using the reasoning of [2], in a MHP of two chromosomes c_a and c_b of n_a and n_b tbs without dispersed paralogy, involving n_{ab} hps, the probability of obtaining exactly k hps in a window W_{ab} of size $l_a \times l_b$ is:

$$p_d(k, l_a, l_b, n_{ab}, n_a, n_b) = \begin{cases} 0, & \text{if } k \leq min(l_a, l_b, n_{ab}) \\ \dfrac{\binom{n_{ab}}{k} \sum_{i=0}^{min(l_a-k, n_{ab}-k)} \binom{n_{ab}-k}{i} \binom{n_a - n_{ab}}{l_a - (k+i)} \binom{n_b - (k+i)}{l_b - k}}{\binom{n_a}{l_a}\binom{n_b}{l_b}}, & \text{otherwise} \end{cases}$$

It is easy to demonstrate that $p_d(1, 1, 1, n_{ab}, n_a, n_b) = \frac{n_{ab}}{n_a \times n_b}$, the density of the MHP. We also have the extreme case $p_d(n_{ab}, n_a, n_b, n_{ab}, n_a, n_b) = 1$. The numerator is the number of ways to fill the chromosomal windows W_a (length l_a tbs) and W_b (length l_b tbs) with tbs from c_a and c_b in order to obtain exactly k hps in W_{ab}. The denominator is the total number of ways to fill W_a and W_b with tbs from c_a and c_b. This denominator is simply the total number of combinations of l_a tbs among the n_a tbs times the total number of combinations of l_b tbs among the n_b tbs. The first term of the numerator $\binom{n_{ab}}{k}$ corresponds to the number of ways to choose the k hps of W_{ab} among the total number of n_{ab} hps. Once these hps are chosen, it remains $l_a - k$ tbs to choose in order to fill W_a. Because there are many ways to choose them, we sum over all the possible combinations. Each of these may involve a different number of "coloured tbs", tbs that have a hp in the MHP (see figure S5). Considering that we choose to put i coloured tbs in W_a, $\binom{n_{ab}-k}{i}$ counts all the possible combinations of i coloured tbs among the $n_{ab} - k$ remaining coloured tbs. $\binom{n_a - n_{ab}}{l_a - (k+i)}$ counts the number of combinations to finish to fill W_a with "grey tbs", tbs that do not have hps in the MHP (see figure S5). Finally the last term, $\binom{n_b - (k+i)}{l_b - k}$ corresponds to the number of ways of choosing the remaining $l_b - k$ tbs in c_b in order to fill W_b, while avoiding choosing the i coloured tbs that would generate a hp in W_{ab} because of the i coloured tbs that we have already placed in W_a. We numerically verified that $p_d(k, l_a, l_b, n_{ab}, n_a, n_b) = p_d(k, l_b, l_a, n_{ab}, n_b, n_a)$.

7 Calculation of $P(sign = s)$

Given that a hp has a sign=$sign$, if we note tb_a and tb_b the two corresponding tbs and $o(\mathsf{tb})$ the orientation of tb, we have:

$$\begin{aligned}
P(sign = +1) &= P(o(\mathsf{tb}_a) = +1)P(o(\mathsf{tb}_b) = +1) \ + \ P(o(\mathsf{tb}_a) = -1)P(o(\mathsf{tb}_b) = -1) \\
P(sign = -1) &= P(o(\mathsf{tb}_a) = -1)P(o(\mathsf{tb}_b) = +1) \ + \ P(o(\mathsf{tb}_a) = +1)P(o(\mathsf{tb}_b) = -1) \\
P(sign = \varnothing) &= P(o(\mathsf{tb}_a) = \varnothing)P(o(\mathsf{tb}_b) = +1) \ + \ P(o(\mathsf{tb}_a) = \varnothing)P(o(\mathsf{tb}_b) = -1) \\
&\quad + P(o(\mathsf{tb}_a) = +1)P(o(\mathsf{tb}_b) = \varnothing) \ + \ P(o(\mathsf{tb}_a) = -1)P(o(\mathsf{tb}_b) = \varnothing) \\
&\quad + P(o(\mathsf{tb}_a) = \varnothing)P(o(\mathsf{tb}_b) = \varnothing)
\end{aligned}$$

$P(o(\mathsf{tb}) = +1)$, $P(o(\mathsf{tb}) = -1)$ and $P(o(\mathsf{tb}) = \varnothing)$ are estimated on c_a and c_b using the frequencies of the orientations of tbs in both chromosomes.

8 Demonstration of the $p_{o,o}$ formula

The probability that k hps form a consistent diagonal is

$$p_{o,o}(k) = \begin{cases} 1, & \text{if } k = 1 \\ p_{slash}(k) + p_{backslash}(k), & \text{otherwise} \end{cases}$$

This probability is equal to the probability to form a consistent slash diagonal or a consistent backslash diagonal. The case $k = 1$ allows an extension of the formula to "diagonals" that contain 1 hp. In this case the intersection of the two probabilities is not null and they cannot be directly summed. In other words, the fact that a "diagonal" of 1 hp can be both a slash and a backslash diagonal if the hp sign is $\varnothing$ is a special case.

9 Explanation of the p_w formula

In a MHP of two chromosomes c_a and c_b of n_a and n_b tbs without dispersed paralogy, involving n_{ab} hps, the probability that in a window W_{ab} of size $l_a \times l_b$ there is at least one consistent diagonal containing at least m hps spaced by gaps $\leq g$ is

$$p_w(m, g, l_a, l_b, n_{ab}, n_a, n_b) = \begin{cases} 0, & \text{if } m > min(n_{ab}, l_a, l_b) \\ \sum\limits_{k=m}^{min(n_{ab}, l_a, l_b)} p_d(k) \sum\limits_{i=m}^{k} p_{g,2\mathrm{D}}(i, g) p_{o,o}(i), & \text{otherwise} \end{cases}$$

Only varying parameters are shown in the right-hand side of the equation in the preceding formula. Since $p_d(k)\ \forall k \in [m, min(n_{ab}, l_a, l_b)]$ are the probabilities of having *exactly* k homologies in a window of size $l_a \times l_b$, we can add these probabilities without removing the probabilities of the intersections. The second sum allows some hps in W_{ab} to not be involved in a consistent diagonal with gaps $\leq g$. If we already know that there is at least m hps in W_{ab}, $\sum\limits_{i=m}^{k} p_{g,2\mathrm{D}}(i, g) p_{o,o}(i)$ is an upper bound for the probability that there are at least m hps forming a consistent diagonal with gaps $\leq g$ in W_{ab}. To be exact we should remove the probabilities of the intersections while summing probabilities. Indeed, the probability of forming a consistent diagonal of 4 hps and the probability of forming a consistent diagonal of 3 hps are dependent since a consistent diagonal of 3 hps is a subset of a consistent diagonal of 4 hps. However removing the probability of the intersection is not trivial and we have therefore chosen an upper bound in order to retain the specificity of the statistical filtering. It is easy to verify that $p_w(m = 1, g = 0, l_a = 1, l_b = 1, n_{ab}, n_a, n_b) = \frac{n_{ab}}{n_a \times n_b}$ whatever the values of n_{ab}, n_a and n_b.

10 Explanation of the passage from a window sampling probability to a whole genome comparison probability

Relying on the reasoning of section 4.2 of [1] we adjust the probability p_w, corresponding to a window sampling scenario, to compute the probability corresponding to a whole genome comparison.

In a MHP of size $n_a \times n_b$ containing n_{ab} hps without dispersed paralogy (see discussion), the probability of finding *at least* one window W_{ab} of size $l_a \times l_b$ containing *at least* one consistent diagonal with gaps $\leq g$ of *at least* m hps can be approximated by:

$$pVal(m, g, l_a, l_b, n_{ab}, n_a, n_b) \simeq 1 - (1 - p_w)^{n_w} \quad (1)$$

where $n_w = \dfrac{n_a n_b}{l_a l_b}$ is the number of windows of width l_a and height l_b in the MHP such that no window overlap with any other window. Still following the reasoning of [1], this last equation is based on an unwarranted assumption that finding clusters in the various n_w windows are independent events.

It should be noted that a linearisation considering that $p_w << 1$ highlights the missing $O(n_a n_b)$ term:

$$pVal(m, g, l_a, l_b, n_{ab}, n_a, n_b) \simeq n_w p_w = \frac{n_a n_b}{l_a l_b} p_w = O(n_a n_b) p_w \quad (2)$$

11 Numerical applications of the p-value formula

In the example of figure S5, the consistant diagonal in W_{ab} has $m = 4$ hps, a width $l_a = 6$ tbs, a height $l_b = 4$ tbs and its maximum gap $g = 2$ tbs is reached on c_a. There are $n_{ab} = 6$ hps in the MHP. c_a contains $n_a = 11$ tbs, and c_b contains $n_b = 8$ tbs. Statistics on the orientations of tbs gives us $P(o(\mathsf{tb}_a) = +1) = \frac{4}{11}$, $P(o(\mathsf{tb}_a) = -1) = \frac{6}{11}$, $P(o(\mathsf{tb}_a) = \varnothing) = \frac{1}{11}$, $P(o(\mathsf{tb}_b) = +1) = \frac{3}{8}$, $P(o(\mathsf{tb}_b) = -1) = \frac{4}{8}$ and $P(o(\mathsf{tb}_b) = \varnothing) = \frac{1}{8}$. A numerical application gives:

$$\begin{aligned}
p_d(k = 4, l_a = 6, l_b = 4, n_{ab} = 6, n_a = 11, n_b = 8) &= 9.7 \times 10^{-3} \\
p_{g,2\mathrm{D}}(k = 4, g = 2, l_a = 6, l_b = 4) &= 1.0 \\
p_{o,o}(k = 4) &= 1.1 \times 10^{-2} \\
p_w(m = 4, g = 2, l_a = 6, l_b = 4, n_{ab} = 6, n_a = 11, n_b = 8) &= 1.1 \times 10^{-4} \\
pVal(m = 4, g = 2, l_a = 6, l_b = 4, n_{ab} = 6, n_a = 11, n_b = 8) &= 3.9 \times 10^{-4}
\end{aligned}$$

$p_{g,2\mathrm{D}}(k = 4, g = 2, l_a = 6, l_b = 4) = 1.0$ because any combination of 4 tbs in W_a will create a chain of 4 hps with gaps $\leq g = 2$. The same applies to tbs in W_b. Thus any cluster is guaranteed to possess gaps lower or equal to 2 in W_{ab} if there is at least 4 hps in W_{ab}. If the cut-off probability α is set to 1×10^{-3}, since the p-value of this consistent diagonal is lower, this consistent diagonal is validated as a significant synteny block. Here it is obvious that for such small diagonal and small chromosomes, accounting for tbs order and tbs orientations is important for the computation of the p-value.

An example of a more common case would be to compute the p-value of a consistent diagonal which has $m = 3$ hps, a width $l_a = 18$ tbs, a height $l_b = 10$ tbs and a maximum gap $g = 10$ tbs. The MHP is characterized by $n_{ab} = 400$ hps, $n_a = 1750$ tbs and $n_b = 2000$ tbs. Usually statistics on genomes give $P(o(\mathsf{tb}_a) = +1) = 0.49$, $P(o(\mathsf{tb}_a) = -1) = 0.49$, $P(o(\mathsf{tb}_a) = \varnothing) = 0.02$, $P(o(\mathsf{tb}_b) = +1) = 0.49$, $P(o(\mathsf{tb}_b) = -1) = 0.49$ and $P(o(\mathsf{tb}_b) = \varnothing) = 0.02$. This time a numerical application gives:

$$\begin{aligned}
p_d(k = 3, l_a = 18, l_b = 10, n_{ab} = 400, n_a = 1750, n_b = 2000) &= 8.6 \times 10^{-7} \\
p_{g,2\mathrm{D}}(k = 3, g = 5, l_a = 18, l_b = 10) &= 0.91 \\
p_{o,o}(k = 3) &= 4.7 \times 10^{-2} \\
p_w(m = 3, l_a = 18, l_b = 10, n_{ab} = 400, n_a = 1750, n_b = 2000) &= 3.7 \times 10^{-8} \\
pVal(m = 3, l_a = 18, l_b = 10, n_{ab} = 400, n_a = 1750, n_b = 2000) &= 7.2 \times 10^{-4}
\end{aligned}$$

Here results show that even a consistent diagonal with very long gaps may be considered as a relevant sb with a cut-off probability α set to 1×10^{-3}. This is possible because tbs order and orientations are considered when assessing the statistical relevance of the sb.

Finally, in a real example, we compare human chromosome Y (hY) to mouse chromosome Y (mY), where PhylDiag, using the CD metric and a $gap_{max} \geq 5$, extracts a consistent diagonal of 3 hps. The maximum gap in this diagonal is $g = 5$ and the sb is contained within a window W_{ab} of size 8×5. hY contains $n_{hY} = 25$ tbs and mY contains $n_{mY} = 16$ tbs. The corresponding MHP contains $n_{hY,mY} = 7$ hps. Statistics on the orientations of tbs gives us $P(o(\mathsf{tb}_{hY}) = +1) = 0.56$, $P(o(\mathsf{tb}_{hY}) = -1) = 0.24$, $P(o(\mathsf{tb}_{hY}) = \varnothing) = 0.20$, $P(o(\mathsf{tb}_{mY}) = +1) = 0.375$, $P(o(\mathsf{tb}_{mY}) = -1) = 0.50$ and $P(o(\mathsf{tb}_{mY}) = \varnothing) = 0.125$ This time a numerical application gives:

$$
\begin{aligned}
p_d(k=3, l_{hY}=8, l_{mY}=5, n_{hY,mY}, n_{hY}, n_{mY}) &= 1.3 \times 10^{-2} \\
p_{g,\mathrm{2D}}(k=3, g=5, l_{hY}=8, l_{mY}=5) &= 1.0 \\
p_{o,o}(k=3) &= 9.2 \times 10^{-2} \\
p_w(m=3, g=5, l_{hY}=8, l_{mY}=5, n_{hY,mY}, n_{hY}, n_{mY}) &= 1.3 \times 10^{-3} \\
pVal(m=3, g=5, l_{hY}=8, l_{mY}=5, n_{hY,mY}, n_{hY}, n_{mY}) &= 1.3 \times 10^{-2}
\end{aligned}
$$

Since the p-value of this consistent diagonal is higher than the cut-off probability $\alpha = 1 \times 10^{-3}$, this diagonal is removed during the statistical validation.

12 Estimation of a recommended maximum gap parameter

As in ColinearScan [3], under the null hypothesis, we assume that homologous tbs are uniformly distributed in chromosomes and we explore the possibility of finding consistent diagonals with gaps $\leq g$ containing m hps by chance. Although this assumption of a uniform distribution is not strictly correct, we consider it to be reasonable here for the purpose of finding a recommended gap_{max}. We consider that the probability of finding consistent diagonals with gaps $\leq g$ containing m hps can be calculated from an average MHP. The average MHP has a width $\overline{n_a}$ (respectively a height $\overline{n_b}$) equal to the weighted mean of the distribution of chromosome lengths of G_a (respectively G_b):

$$\overline{n_a} = \sum_{c_a \in G_a} w_{c_a} n_{c_a} \tag{3}$$

where $w_{c_a} = \frac{n_{c_a}}{\sum\limits_{c_a \in G_a} n_{c_a}}$ is the weight given to the length n_{c_a} (in tbs) of c_a. The average MHP is designed in order to have the same density as the the whole genome comparison of G_a with G_b. The density of the whole genome comparison is:

$$\theta_{G_a Gb} = \frac{n_{G_a G_b}}{n_{G_a} n_{G_b}}, \tag{4}$$

where $n_{G_a G_b}$ is the total number of hps in the whole genome comparison of G_a with G_b and n_{G_x} is the total number of tbs in G_x, thus the number of hps in the average MHP is:

$$\overline{n_{ab}} = \overline{n_a}\, \overline{n_b} \times \theta_{GaGb}, \tag{5}$$

For many gaps values (g), a numerical computation of $pVal(m, g, l_a, l_b, \overline{n_{ab}}, \overline{n_a}, \overline{n_b})$ with $l_a = l_b = (m-1)g+m$ are performed. The recommended gap_{max} value is defined as the lowest gap g that returns a p-value higher than the target probability P_{target}. m and P_{target} are fixed by the user. Default values are $m = 2$ and $P_{target} = 0.01$. For instance, when comparing the human (S_h) to the mouse (S_m) (Ensembl database v72) we have: $n_h = 18560$ tbs, $n_m = 18934$ tbs and $n_{hm} = 18236$ hps. The average MHP is characterised by $\overline{n_a} = 980$, $\overline{n_b} = 1052$ and $\overline{n_{ab}} = 53$. With the default values of m and P_{target}, the recommended maximum gap value is 5.

It should be noted that it is not because we use a gap_{max} parameter of 5 that we will statistically validate all consistent diagonals with gaps up to 5. When a consistent diagonal is found it undergoes the statistical validation that depends on the value of the probability threshold α (default value is 1×10^{-3}) and also on the characteristics (density of hps, dimensions, ...) of the MHP of the current pairwise comparison of chromosomes that may differ from the characteristics of the average MHP. Here is an example. Still in the comparison of the human genome with the mouse genome, we consider the comparison of the human X chromosome (c_{hX})

with the mouse X chromosome (c_{mX}). In the MHP of this comparison, PhylDiag (using the recommended $gap_{max} = 5$) finds a consistent diagonal of 2 hps characterized by a maximum gap $g = 3$ and a window W_{ab} of size 3×5. Given that $n_{hX} = 735$ is the number of tbs in c_{hX} and $n_{mX} = 726$ is the number of tbs in c_{mX} and $n_{hX,mX} = 690$ is the number of hps involved in the comparison of c_{hX} with c_{mX}, the p-value of the putative synteny block is $pVal(2, 3, 3, 5, n_{hX,mX}, n_{hX}, n_{mX}) = 0.63$. Since this p-value is $> \alpha = 1 \times 10^{-3}$ the putative synteny block is rejected. This is not surprising since $\frac{n_{hX,mX}}{n_{hX} n_{mX}} = 1.3 \times 10^{-3} >> \frac{\overline{n_{h,m}}}{\overline{n_h}\ \overline{n_m}} = 5.1 \times 10^{-5}$.

13 Simulator

Our simulator first designs an ancestral genome G_{anc} with a user defined number of genes and chromosomes. The length of chromosomes in G_{anc} are expressed in number of genes, and are determined randomly. Simulated evolution gives rise to the two extant genomes G_a and G_b of two extant species. The simulator performs genic events, which include de novo gene births, deletions, duplications (either tandem or dispersed), and genomic rearrangements, which include chromosome fusions and fissions, segmental translocations or segmental inversions.

Inversions and translocations involve a chromosomal segment. Each time a translocation or an inversion occurs, a chromosome is chosen with a frequency that depends on its length (i.e. the longer a chromosome, the higher the chance that it will be chosen). The length of the rearranged segment is chosen as a proportion of the chromosome length in a density function represented on figure S6 obtained from a modification of the von Mises probability distribution. If it is a translocation, the insertion position is chosen with a uniform

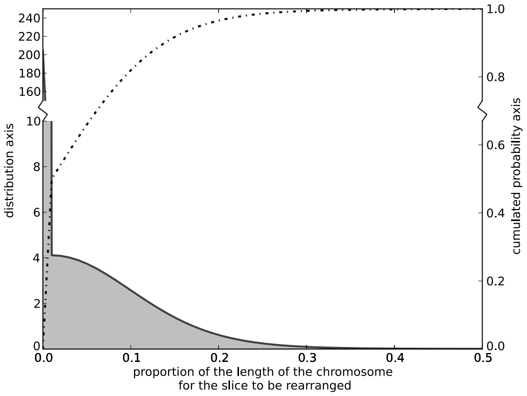

Figure S6: **Theoretical distribution of the lengths of rearranged chromosomal segments.** The length of a rearranged chromosomal segment is calculated as a proportion of the length of the departure chromosome. For each proportion of the length on the X-Axis, the black curve represents the probability density of choosing a segment of this length. The left Y-axis reports values of the probability density. The doted line represents the cumulated probability, and its value is reported on the right Y-axis.

probability on all possible insertion positions. A full description of the simulator will be published elsewhere (Muffato et al. in preparation).

The evolutionary scenario is calibrated so as to fit the known evolution of the human and the mouse genome from the Euarchontoglire genome (G_{anc}). Based on phylogenetic gene tree reconstructions from Ensembl Compara version 72, the Euarchontoglire genome possessed at least 21806 genes that evolved during approximately 90 million years into the human genome on the one hand and into the mouse genome on the other hand. In each simulation, the ancestral Euarchontoglire genome is populated with the same 21806 genes distributed into 20 chromosomes, but in a different random order. The extant human genome contains 20172 genes and the mouse genome contains 22542 genes. According to the forest of gene trees stemming from Euarchontoglire, 3836 gene deletions, 821 de novo gene births, 1381 gene duplications with 791 tandem duplications (57%) and 590 dispersed duplications took place in the human lineage. Similarly, 4060 gene deletions, 1658 de novo gene births and 3138 gene duplications with 1950 tandem duplications (62%) and 1188 dispersed duplications took place in the mouse lineage. We calibrated the rates of rearrangements on

each branch, starting from known rates [4] and optimised to visually reproduce the distribution of genes between the real mouse and human genomes (table S1 and figure S7). Of note, we aim here at simulating the evolution of the human and mouse genome in a reasonably realistic way. A proper modelling of this process is out of the scope of this study, so long as simulated data make it possible to compare different methods to identify synteny blocks.

**Rates**	E $\rightarrow$ H	E $\rightarrow$ M
Duplication	15	35
Tandem Dup	60%	60%
Deletion	43	45
Apparition	9	18
Inversion	0.8	2.6
Translocation	0.22	0.48
Fusion	0.26	0.26
Fission	0.26	0.26

Table S1: **Rates of the different events on each of the two branches.** Rates are in number of events per million years. E $\rightarrow$ H is the branch from Euarchontoglire to Human and E $\rightarrow$ M is the branch from Euarchontoglire to Mouse. Each branch lasts 90 million years. “Tandem Dup” is the proportion of tandem duplications among duplications.

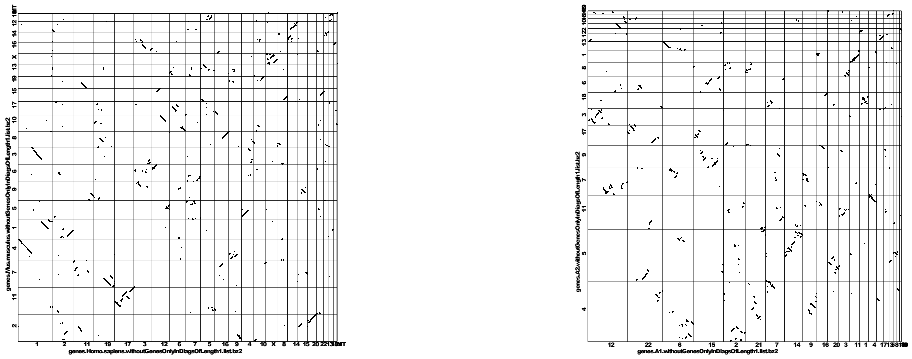

(a) Homology matrix of the whole-genome comparison between the real human genome (Y-axis) and the real mouse genome (X-axis)

(b) Homology matrix of the whole-genome comparison between a simulated human genome (Y-axis) and a simulated mouse genome (X-axis)

Figure S7: **Homology matrices of whole genome comparisons, with real genomes and simulated genomes.**

14 Influence of genic events and chromosomal rearrangements on the MHP and comparison between the DPD and the MD

Figure S8 shows how different events disturb or not the linearity of synteny blocks. Based on figure S8, since merging diagonals with the DPD metric attributes more importance to linearity, chosing the DPD metric will allow more small inversions within sbs gaps while considering that genic/segmental indels and wrong annotations break the synteny more easily than with the MD metric. Conversely, merging diagonals with the MD metric gives priority to lateral directions and this allows more small genic/segmental indels and annotation errors within sbs gaps and considers that inversions break the synteny more easily than with the DPD metric.

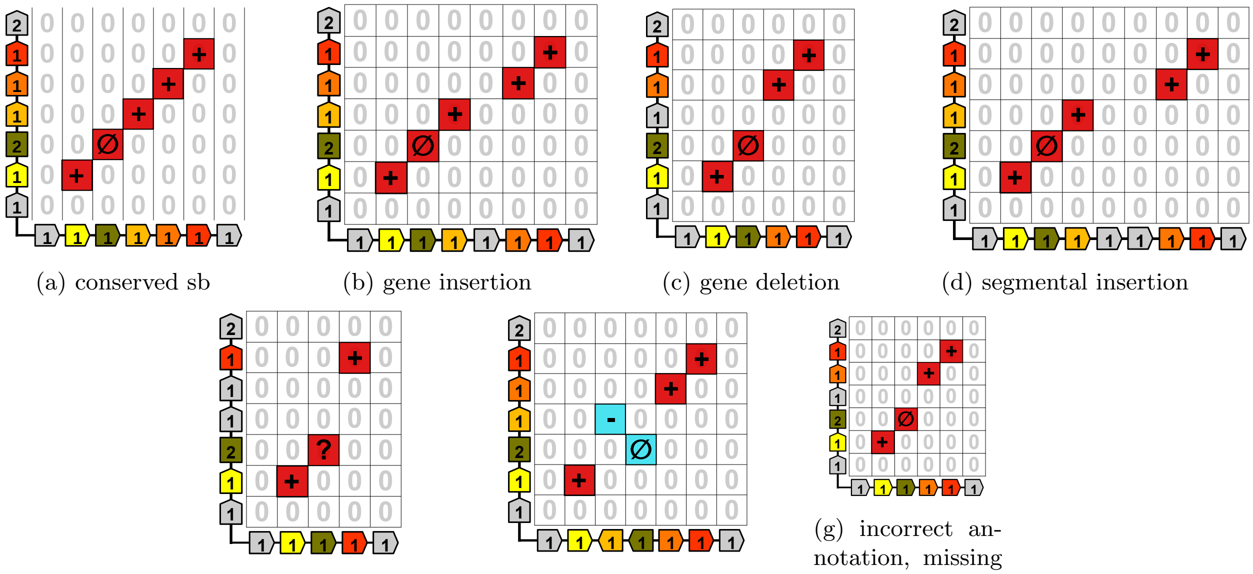

(a) conserved sb (b) gene insertion (c) gene deletion (d) segmental insertion

(e) segmental deletion (f) segmental inversion (g) incorrect annotation, missing one gene

Figure S8: **Examples of evolutionary scenarios of a synteny block.** Inversion events create a gap in the sb and do not change the linearity of the sb, whereas gene indels, segmental indels and annotation errors affect the linearity of the sb.

List of abbreviations used

Acronyms

sb (plural sbs)	Synteny Block
CAR	Contiguous Ancestral Region
tb (plural tbs)	Tandem Block
hp (plural hps)	Homology Pack
MH	Matrix of Homologies
MHP	Matrix of Homology Packs
ED	Euclidean Distance metric
CD	Chebyshev Distance metric
MD	Manhattan Distance metric
DPD	Diagonal Pseudo Distance metric

Vocabulary

synteny block (sb)	an ordered sequence of close oriented genes that is conserved in several species
gene specific to one lineage	a gene that did not exist in the ancestor and that appeared along a lineage from the ancestor to one extant species
tandem block (tb)	a contiguous sequence of tandem duplicates
homology pack (hp)	a pack of homologies corresponding to a homology between two tandem blocks
sign of a hp	a value that indicates whether or not the two corresponding homologous tandem blocks are in the same orientation, the opposite orientation or if at least one tandem block has an *unknown* orientation
gap between two tbs	the number of tbs between them
distance between two tbs	the gap between them *minus one*
distance metric	a metric that is used to calculate a distance between two points in a 2D array
distance between two hps	given a distance metric, the distance between the two points corresponding to the two hps in the MHP
gap between two hps	the distance between them *plus one*
chain	a set of tbs spaced by gaps $\leq gap_{max}$
cluster	a set of hps spaced by gaps $\leq gap_{max}$
diagonal	a diagonal of hps in a MH or a MHP, it may be interpreted as a cluster with a constraint on gene order and gene orientations
strict diagonal	a diagonal of hps with no gaps between hps
slash diagonal	a type of diagonal that goes from bottom-left to top-right
backslash diagonal	a type of diagonal that goes from top-left to bottom-right
consistent diagonal	a diagonal of hps with signs consistent with the diagonal type
putative synteny block	a consistent diagonal that may be a synteny block if it passes the statistical validation

Notations

S_a	a Species
$\mathsf{CA}(S_a, S_b)$	a Common Ancestor of S_a and S_b
$\mathsf{LCA}(S_a, S_b)$	the Last Common Ancestor of S_a and S_b
G_a	the Genome of S_a
c_a	a Chromosome of G_a
g	an instance of Gene
g_a	an oriented gene of G_a
N_a	number of genes in c_a
tb	an instance of Tandem Block
tb_a	an oriented tb of G_a
n_a	number of tbs in c_a
$c_a[i_s \to i_e]$	sub-list of c_a that goes from the ${i_s}^{th}$ index to the ${i_e}^{th}$ index of the chromosome c_a
$o(\mathsf{tb})$	orientation of tb relatively to the reference orientation of the chromosome containing tb, either $+1$,-1 or $\varnothing$
$\varnothing$	null value or *unknown* value
$\mathsf{tb}_{a,i} \bullet \mathsf{tb}_{b,j}$	comparison of the orientation of the ith tb of the chromosome c_a with the orientation of the jth tb of the chromosome c_b
$x \,\mathcal{H}\, y$	x is in a homology relation with y
N_{ab}	number of homologies in the MH of the two chromosomes c_a and c_b
hp	an instance of Homology Pack
n_{ab}	number of hps in the MHP of the two chromosomes c_a and c_b
$\theta_{c_a c_b}$	the density of the comparison of the two chromosomes c_a and c_b
$\theta_{G_a G_b}$	the density of the whole genome comparison of the two genomes G_a and G_b
$s(\mathsf{hp})$	sign of hp, either $+1$, -1 or $\varnothing$
$d_{\mathsf{DM}}((x_1, y_1), (x_2, y_2))$	distance between the point (x_1, y_1) and the point (x_2, y_2) using the distance metric DM
$\mathsf{LCAg}(\mathsf{tb}_a, \mathsf{tb}_b)$	the Last Common Ancestral Gene of tb_a and tb_b
$\mathfrak{M}_{rows,cols}$	set of matrices of size $rows \times cols$
$\mathsf{M}[i, j]$	element of the ith row and the jth column of the matrix M
$M[i_s \to i_e, j_s \to j_e]$	sub-matrix of M that goes from the ${i_s}^{th}$ row to the ${i_e}^{th}$ row and from the ${j_s}^{th}$ column to the ${j_e}^{th}$ column
sb	an instance of Synteny Block
$\overline{n_a}$	number of tbs on the c_a chromosome of the average MHP
$\overline{n_{ab}}$	number of hps in the average MHP

References

[1] D. Durand and D. Sankoff. Tests for gene clustering. *Journal of computational biology : a journal of computational molecular cell biology*, 10(3-4):453–82, Jan. 2003.

[2] N. Raghupathy, R. Hoberman, and D. Durand. Two plus two does not equal three: statistical tests for multiple genome comparison. *Journal of bioinformatics and computational biology*, 6(1):1–22, Feb. 2008.

[3] X. Wang, X. Shi, Z. Li, Q. Zhu, L. Kong, W. Tang, S. Ge, and J. Luo. Statistical inference of chromosomal homology based on gene colinearity and applications to arabidopsis and rice. *BMC Bioinformatics*, 7:447, Oct. 2006. PMID: 17038171 PMCID: PMC1626491.

[4] H. Zhao and G. Bourque. Recovering genome rearrangements in the mammalian phylogeny. pages 934–942, 2009.
